# Association of oral bisphosphonates with cardioembolic ischemic stroke: a nested case-control study

**DOI:** 10.3389/fphar.2023.1197238

**Published:** 2023-05-26

**Authors:** Sara Rodríguez-Martín, Diana Barreira-Hernández, Ramón Mazzucchelli, Miguel Gil, Alberto García-Lledó, Laura Izquierdo-Esteban, Ana Pérez-Gómez, Antonio Rodríguez-Miguel, Francisco J. De Abajo

**Affiliations:** ^1^ Department of Biomedical Sciences (Pharmacology), University of Alcalá (IRYCIS), Alcalá de Henares, Spain; ^2^ Rheumatology Department, University Hospital “Fundación Alcorcón”, Madrid, Spain; ^3^ Division of Pharmacoepidemiology and Pharmacovigilance, Spanish Agency on Medicines and Medical Devices, Madrid, Spain; ^4^ Department of Cardiology, University Hospital “Príncipe de Asturias”, Alcalá de Henares, Spain; ^5^ Department of Medicine, University of Alcalá, Alcalá de Henares, Spain; ^6^ Department of Neurology, Stroke Unit, University Hospital “Príncipe de Asturias”, Alcalá de Henares, Spain; ^7^ Department of Rheumatology, University Hospital “Príncipe de Asturias”, Alcalá de Henares, Spain; ^8^ Clinical Pharmacology Unit, Universty Hospital “Príncipe de Asturias”, Alcalá de Henares, Spain

**Keywords:** bisphosphonates, ischemic stroke (IS), cardioembolic stroke, atrial fibrillation, antiresorptive agents, osteoporosis

## Abstract

**Background:** Bisphosphonates have been reported to increase the risk of atrial fibrillation. Therefore, it is conceivable that they may increase the risk of cardioembolic ischemic stroke (IS). However, most epidemiological studies carried out thus far have not shown an increased risk of IS, though none separated by the main pathophysiologic IS subtype (cardioembolic and non-cardioembolic) which may be crucial. In this study, we tested the hypothesis that the use of oral bisphosphonates increases specifically the risk of cardioembolic IS, and explored the effect of treatment duration, as well as the potential interaction between oral bisphosphonates and calcium supplements and anticoagulants.

**Methods:** We performed a case-control study nested in a cohort of patients aged 40–99 years, using the Spanish primary healthcare database BIFAP, over the period 2002-2015. Incident cases of IS were identified and classified as cardioembolic or non-cardioembolic. Five controls per case were randomly selected, matched for age, sex, and index date (first recording of IS) using an incidence-density sampling. The association of IS (overall and by subtype) with the use of oral bisphosphonates within the last year before index date was assessed by computing the adjusted odds ratios (AOR) and their 95% CI using a conditional logistic regression. Only initiators of oral bisphosphonates were considered.

**Results:** A total of 13,781 incident cases of IS and 65,909 controls were included. The mean age was 74.5 (SD ± 12.4) years and 51.6% were male. Among cases, 3.15% were current users of oral bisphosphonates, while among controls they were 2.62%, yielding an AOR of 1.15 (95% CI:1.01–1.30). Of all cases, 4,568 (33.1%) were classified as cardioembolic IS (matched with 21,697 controls) and 9,213 (66.9%) as non-cardioembolic IS (matched with 44,212 controls) yielding an AOR of 1.35 (95% CI:1.10–1.66) and 1.03 (95% CI: 0.88–1.21), respectively. The association with cardioembolic IS was clearly duration-dependent (AOR≤1 year = 1.10; 95% CI:0.82–1.49; AOR>1–3 years = 1.41; 95% CI:1.01–1.97; AOR>3 years = 1.81; 95% CI:1.25–2.62; *p* for trend = 0.001) and completely blunted by anticoagulants, even in long-term users (AOR>1 year = 0.59; 0.30–1.16). An interaction between oral bisphosphonates and calcium supplements was suggested.

**Conclusion:** The use of oral bisphosphonates increases specifically the odds of cardioembolic IS, in a duration-dependent manner, while leaves materially unaffected the odds of non-cardioembolic IS.

## 1 Introduction

Bisphosphonates, the first-line drugs for patients at risk of osteoporotic fractures (Lems et al., 2017), have been reported to increase the risk of atrial fibrillation (AF) (Black et al., 2007; Sharma et al., 2013; Kim et al., 2015; Park and Ko, 2022), although not all studies provided consistent results (Cummings et al., 2007; Reid et al., 2018; Rodríguez et al., 2020). If this association were causal, it would be conceivable that bisphosphonates increase the risk of cardioembolic ischemic stroke (IS) as well; however, the evidence is scarce. In two meta-analyses of randomized clinical trials (Sharma et al., 2013; Kim et al., 2015), no association was found between bisphosphonates and stroke (OR: 0.99; 95% CI:0.82–1.19 and 0.92; 95% CI:0.68–1.26, respectively), though the number of events were rather small in most trials, and none had a duration longer than 36 months. Interestingly, in the HORIZON-PFT trial, in which an extension from three to 6 years with zoledronic acid was compared to placebo (analysis not included in the aforementioned meta-analyses), a quasi-significant increased risk of stroke was found (3.1% vs 1.5%; *p* = 0.06) (Black et al., 2012), which suggests a duration-dependent effect that may not be captured by short-term trials. Also, a number of observational studies has been carried out so far and all (Christensen et al., 2011; Kang et al., 2013; Sing et al., 2018; Asghar et al., 2019; Rodríguez et al., 2020) but one (Vestergaard et al., 2011) found no increased risk of stroke. Unfortunately, most did not differentiate between ischemic and hemorrhagic stroke and, among those focusing on ischemic stroke (IS), none distinguished between cardioembolic and non-cardioembolic subtypes, which may be crucial, as bisphosphonates may increase the risk of the former, but not necessarily the risk of the latter.

Bisphosphonates are frequently used with calcium supplements (CaS), which have been reported to increase the risk of thrombotic events (Bolland et al., 2011), including IS (de Abajo et al., 2017). Thus, the potential interaction between bisphosphonates and CaS is of special interest, in particular if we take into account that their concomitant use may further alter the calcium homeostasis in atrial cardiomyocites observed with bisphosphonates (Kemeny-Suss et al., 2009). As far as we know, such interaction has not been studied yet.

This study was carried out to test in real-life conditions the hypothesis that the use of oral bisphosphonates (oBs) increases specifically the risk of cardioembolic IS, in particular after long-term exposure, and to explore a potential interaction with CaS and anticoagulants. Under this hypothesis, non-cardioembolic IS could be envisioned as a negative control outcome.

## 2 Patients and methods

### 2.1 Study design and data source

A nested case-control study was performed using data from BIFAP (Base de datos para la Investigación Farmacoepidemiológica en el Ámbito Público), a Spanish primary care database which includes information related to medical care within the Spanish national health system prospectively recorded by the primary care physicians (PCPs). The information is fully anonymized and includes sociodemographic and life-style data of patients, records on clinical events, results of laboratory and complementary diagnostic tests, as well as all outpatient prescriptions filled by the PCPs by his/her own or at the request of specialists. Clinical events are recorded using the International Classification of Primary Care, version 2 (ICPC-2) or the International Classification of Diseases, version 9, Clinical Modification (ICD-9-CM), depending on the region. All prescriptions written by the PCPs are recorded including product name, quantity, dosing regimens, indication and date of prescription (Maciá-Martínez et al., 2020). The information in the database is enriched with free text annotations of the PCPs. The BIFAP population reflects the population receiving healthcare in Spain (Maciá-Martínez et al., 2020). The 2016 version, which we used for the present study, included data from 7.6 million patients, with an average of 5.1 years of follow-up (totaling 38.8 million person-years), from 9 different Spanish regions (out of 17). BIFAP has been extensively validated through multiple pharmacoepidemiologic studies in different areas (Maciá-Martínez et al., 2020), including cardiovascular and cerebrovascular events (Rodríguez-Martín et al., 2019; Alqdwah-Fattouh et al., 2022; Rodríguez-Martín et al., 2022).

### 2.2 Selection of patients

We first identified a cohort of patients aged 40–99 years registered in the database for at least 1 year during the study period 2002-2015 and without any type of cancer or stroke history (antecedents of transient ischemic attacks (TIA) were allowed). Although, bisphosphonates are prescribed to cancer patients, we decided to exclude these patients because they receive many hospital-dispensed drugs that are not recorded in the database; additionally, patients with cancer may have a limited life expectancy. The first day patients met all criteria mentioned above was considered the “start date”. Then, subjects were followed-up until one of the following events occurred: an incident stroke (any type), 100 years old, cancer, death, or end of the study period, whichever came first. All potential stroke cases were identified using ICPC-2 codes K90 (stroke), as well as ICD-9-CM codes 434. x1 and 436 (cerebrovascular diseases) and the free text associated with the diagnosis. Potential cases of hemorrhagic stroke were excluded, retaining only potential cases of ischemic or unspecified stroke. These cases were grouped into homogeneous sets in terms of available information, and a random sample from each group was manually reviewed independently by two investigators blinded to drug exposures; discrepancies were solved by the entire research group (including a neurologist and a cardiologist). After completing the manual review, the final positive predictive value reached was 87.1%. The date on which the diagnosis of stroke was first recorded was considered the index date.

To identify the most probable pathophysiological subtype of IS, cardioembolic and non-cardioembolic (including in this category large artery atherosclerosis infarct, small vessel occlusion–like lacunar stroke- and stroke of undetermined cause), we checked the PCP’s annotations in the free text associated with the diagnosis before and within 3 months after the event, searching for texts of “cardioembolic,” “atrial fibrillation,” or prescriptions for oral anticoagulants (OACs). These three items were used as the main criteria for supporting a cardioembolic stroke (74.1% of cases met at least two of them) (see Supplementary Methods). Additionally, “mitral valve prosthesis,” or “mitral stenosis,” and use of class IC or III antiarrhythmics were used as complementary criteria provided that at least one of the main criteria was present. Cases not fulfilling these criteria and those including texts such as “atherothrombotic,” “lacunar” or related terms, were considered as non-cardioembolic. Strokes of unusual cause (e.g., vasculitis, dissection, consumption of toxic substances) were excluded from all analyses.

For each case, five controls were randomly selected from the study cohort matched by age, sex, and index date following a risk-set sampling. This sampling is proportional to the person time at risk of patients and makes the odds ratio obtained in the case-control analysis an unbiased estimate of the incidence rate ratio (Rothman et al., 2013).

With a view to focus on oBs initiators, patients with recorded prescriptions of these drugs prior to the starting date were excluded from both cases and controls. After that, a few cases and controls became unmatched and were excluded.

### 2.3 Exposure definition

The drugs of interest were oBs, the active ingredients available in Spain being alendronic acid (ATC: M05BA04 and M05BB03), ibandronic acid (M05BA06), risedronic acid (M05BA07) and etidronic acid (M05BA01). Zoledronic acid was not included as it is only delivered through hospital pharmacies and BIFAP has no accurate recording of these drugs. All bisphosphonates were grouped within the same class, but an analysis by individual drugs was also performed.

Patients were classified into three different categories based on the time elapsed from the end of the last recorded prescription to the index date: (1) “current users” when the last prescription ended within 365 days before the index date; and (2) “past users” when the last prescription ended more than 365 days before the index date; and (3) “nonusers” when there were no prescriptions of oBs recorded before the index date. The last category was used as the reference in the analysis. In some analysis, as indicated, past users and nonusers were collapsed in one category to increase the statistical power. The use of CaS was categorized in the same manner. For other drugs, the category “current use” was defined using a shorter interval (30 days before the index date), as a residual effect is not expected for them. Accordingly, “past use” was defined for those drugs when the last prescription ended more than 30 days before the index date.

Treatment duration of oBs among current users was calculated by summing up the duration of prescriptions given consecutively (allowing a gap of 90 days between the end of one prescription and the start of the next one). Patients were grouped in two categories (up to 1 year and more than 1 year) or, when numbers permitted, in three (up to 1 year, between one and 3 years and more than 3 years).

### 2.4 Covariables and potential confounding factors

To adjust for confounding we considered the following variables using expert criteria: (1) number of PCP visits in the last year (an indirect comorbidity indicator); (2) life-style factors: smoking, alcohol dependence and body mass index (BMI); (3) comorbidities (recorded before the index date): TIA, ischemic heart disease, including history of acute myocardial infarction (AMI) or angina pectoris (recorded as such and/or use of nitrates), venous thromboembolism, heart failure, peripheral artery disease, hypertension, diabetes (recorded as such, and/or use of glucose-lowering drugs), dyslipidemia (recorded as such, and/or use of lipid-lowering drugs), hyperuricemia (asymptomatic or within a diagnosis of gout), chronic obstructive pulmonary disease, rheumatoid arthritis, and chronic renal failure; and (4) use of the following drugs within the last 30 days before index date: antiplatelet agents, beta-blockers, alpha blockers, angiotensin-converting enzyme inhibitors, angiotensin II receptor antagonists, calcium-channel blockers, diuretics, paracetamol, metamizole, non-steroidal anti-inflammatory drugs, corticosteroids, opioids, calcium supplements (with or without vitamin D), other drugs used for osteoporosis (hormonal replacement therapy, estrogen receptor modulators, strontium ranelate, calcitonin, denosumab, and teriparatide), proton pump inhibitors and H2-receptor antagonists.

According to our causal model (Supplementary Figure S1), AF was considered an intermediate variable in the causal pathway between bisphosphonate use and cardioembolic IS and, thus, not included among potential confounders. Due to the high correlation with AF, we did not consider the use of OACs as a confounder, either. However, we analyzed the potential interaction of oBs with AF and use of anticoagulants through specific stratified analyses. For some analyses, patients were grouped according to the background vascular risk, as follows: (1) established vascular disease: patients with a history of ischemic heart disease (AMI or angina pectoris), heart failure, TIA, peripheral arterial disease or diabetes any time before the index date; (2) vascular risk factors: subjects with a record of hypertension, dyslipidemia, chronic renal failure, current smoking, or body mass index ≥30 kg/m2 before the index date with none of the conditions mentioned in the first point; (3) no recorded vascular risk factors or diseases. We also assigned to each patient a CHA2DS2-VASc score.

### 2.5 Statistical analysis

The rationale of the present study is based on the fact that oBs may exhibit different effects on cardioembolic and non-cardioembolic IS. Thus, all analyses have been performed using three outcomes: 1) IS of any type; 2) cardioembolic IS; and 3) non-cardioembolic IS.

The association of oB use with the outcomes of interest was assessed by computing the odds ratio (OR) and its corresponding 95% confidence intervals (95% CI) through a conditional logistic regression. Crude ORs were computed first including oB use as the only explanatory variable, and then the adjusted odds ratios (AORs) by adding all potential confounders mentioned in the previous section. We also assessed the potential interaction of oBs use with age (younger than 70 years and 70 years or older), sex, background vascular risk (established vascular diseases, risk vascular factors, no vascular risk), CHA2DS2-VASc (less than three or greater), AF, use of OACs, use of antiplatelet drugs and use of CaS, by computing the AORs across the strata built with the different categories of the potential interacting variable and comparing them with the interaction test described by Altman and Bland (multiplicative scale) (Altman and Bland, 2003). When the potentially interacting variable was not a matching factor, we performed an unconditional logistic regression (including the matching variables in the model) as the conditional logistic regression provided unstable estimates. The interaction with CaS was also assessed in the additive scale; to that end, we built a variable with four categories: 1) non-use/past use of oBs and non-use/past use of CaS; 2) current use of oBs without current use of CaS; 3) current use of CaS without current use of oBs; and 4) current use of both oBs and CaS. A sub-categorization by oB duration (≤1 year; >1 year) and type of CaS (with or without vitamin D) was also undertaken.

Two variables had missing values, BMI (34.8%) and smoking (49.4%). To address this, multiple imputation by chained equations (MICE) models were run in all analyses.

We performed two sensitivity analyses: 1) joining current and past users of oBs (“ever users”); and 2) adding prevalent users of oBs.

The results were considered statistically significant when *p* < 0.05. All analyses were performed with STATA/MP 17 (StataCorp. College Station, TX, US).

## 3 Results

**FIGURE 1:**
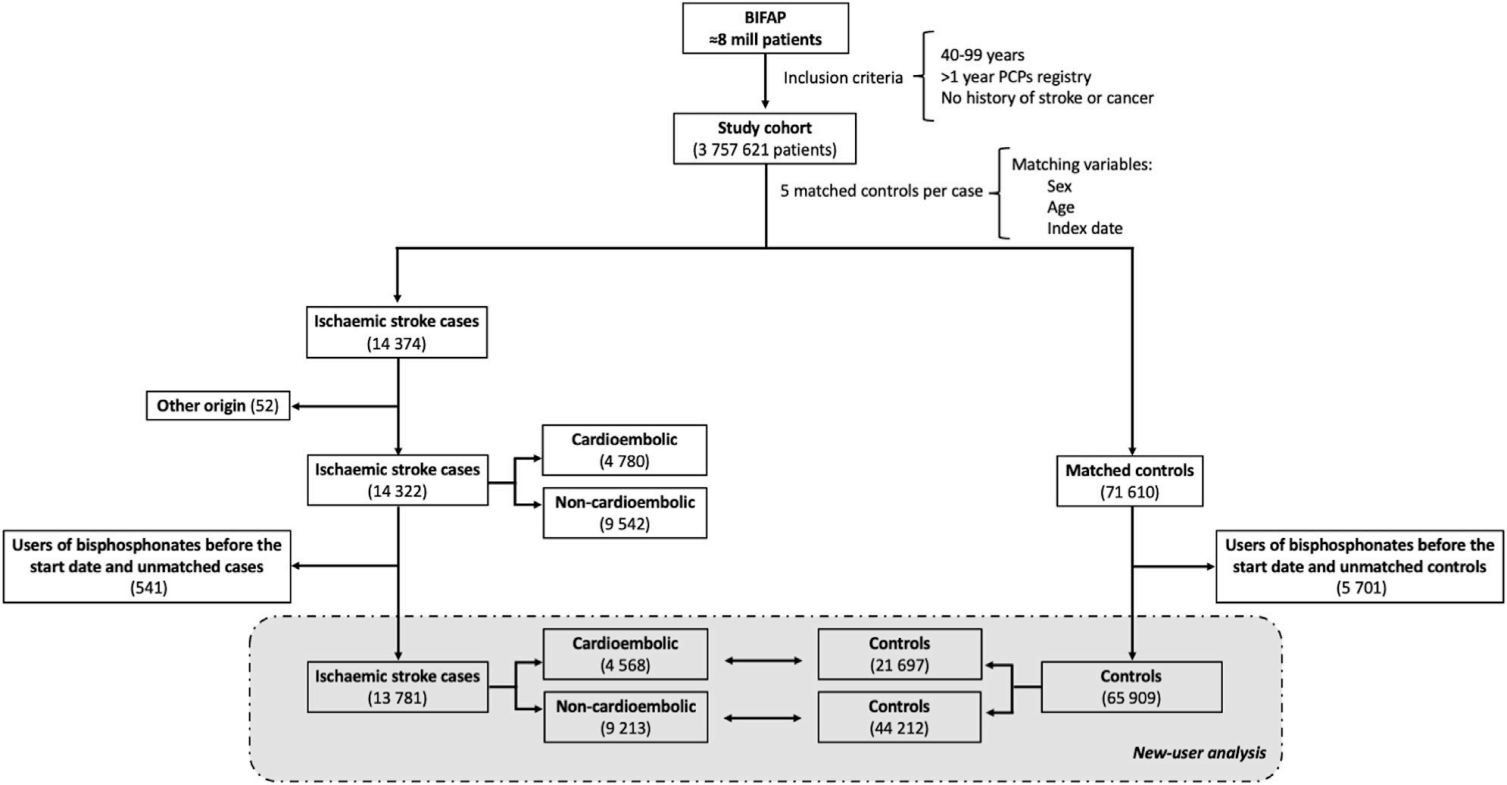
Flowchart of patient selection. Abbreviations: PCP, primary care physician.

From a study cohort of 3,757,621 patients (18,151,030 person-years), we selected 14,374 subjects as valid cases of IS. After excluding prevalent users of oBs and unmatched cases, we retained 13,781 IS cases, 9,213 (66.9%) classified as non-cardioembolic IS and 4,568 (33.1%) as cardioembolic IS (Figure 1). The average follow-up time for cases was 4.2 years (standard deviation -SD-: ±3.2 years).

A total of 65,909 matched controls (mean 4.8 per case) were randomly selected from the study cohort showing a mean (±SD) follow-up time of 3.7 years (±3.0 years). Characteristics of cases and controls at index-date are described in Table 1 for all IS; Supplementary Table S1; Supplementary Table S2 for cardioembolic and non-cardioembolic IS, respectively. Foreseeably, cases presented a higher prevalence of comorbidities than controls, notably those related to the vascular system, as well as a higher proportion of use of OACs, antiplatelets, and different antihypertensive drugs. The mean score ( ± SD) of the CHA2DS2-VASc was 3.07 ( ± 1.62) for cases and 2.69 ( ± 1.57) for controls (*p* < 0.001).

**TABLE 1:** Characteristics of ischemic stroke cases and matched controls.

	Cases (%) N = 13781	Controls (%) N = 65909	Non-adjusted OR^a^ (95% CI)
Age (years); mean (±SD)	74.5 (±12.4)	74.2 (±12.5)	-
Men	7122 (51.68)	35335 (53.61)	-
Visits (last 12 months)			
*Up to 5*	2451 (17.79)	18991 (28.81)	1 (Ref.)
*6–15*	5115 (37.12)	24964 (37.88)	1.67 (1.58–1.76)
*16–24*	3208 (23.28)	12017 (18.23)	2.27 (2.13–2.41)
*25+*	3007 (21.82)	9937 (15.08)	2.67 (2.50–2.84)
Smoking			
*Never smoking*	4506 (32.70)	21318 (32.34)	1 (Ref.)
*Current smoker*	2238 (16.24)	7715 (11.71)	1.51 (1.42–1.61)
*Past smoker*	950 (6.89)	3637 (5.52)	1.34 (1.24–1.46)
*Unknown*	6087 (44.17)	33239 (50.43)	0.88 (0.85–0.92)
Alcohol dependence	412 (2.99)	1051 (1.59)	1.98 (1.76–2.23)
BMI kg/m^2^			
*Up to 24.9*	1918 (13.92)	8913 (13.52)	1 (Ref.)
*25–29*	4083 (29.63)	19183 (29.11)	1.00 (0.94–1.06)
*30–34*	2458 (17.84)	10928 (16.58)	1.05 (0.98–1.12)
*35–39*	724 (5.25)	2803 (4.25)	1.19 (1.09–1.32)
*40+*	228 (1.65)	726 (1.10)	1.46 (1.25–1.71)
Unknown	4370 (31.71)	23356 (35.44)	0.88 (0.82–0.93)
Transient ischemic attack	765 (5.55)	1466 (2.22)	2.60 (2.37–2.85)
Ischemic heart disease			
*Acute myocardial infarction*	854 (6.20)	2383 (3.62)	1.88 (1.73–2.04)
*Angina pectoris^b^*	1239 (8.99)	4161 (6.31)	1.53 (1.43–1.64)
Venous thromboembolic disease	300 (2.18)	1078 (1.64)	1.33 (1.17–1.51)
Heart failure	1026 (7.45)	2946 (4.47)	1.74 (1.61–1.87)
Atrial fibrillation	2012 (14.60)	4940 (7.50)	2.16 (2.04–2.28)
Peripheral artery disease	685 (4.97)	1652 (2.51)	2.09 (1.91–2.29)
Hypertension	8599 (62.40)	34632 (52.55)	1.54 (1.48–1.60)
Diabetes^c^	3972 (28.82)	12599 (19.12)	1.72 (1.65–1.80)
Dyslipidemia^d^	6083 (44.14)	25671 (38.95)	1.24 (1.20–1.29)
Hyperuricemia			
*Asymptomatic*	1092 (7.92)	4784 (7.26)	1.12 (1.05–1.21)
*Gout*	697 (5.06)	2646 (4.01)	1.32 (1.21–1.44)
COPD	1196 (8.68)	4946 (7.50)	1.20 (1.13–1.29)
Rheumatoid arthritis	99 (0.72)	493 (0.75)	0.94 (0.76–1.17)
Chronic renal failure	787 (5.71)	2467 (3.74)	1.56 (1.44–1.70)
Background vascular risk			
*No risk factors/diseases*	1910 (13.86)	14946 (22.68)	1 (Ref.)
*Risk factors only*	5603 (40.66)	30678 (46.55)	1.46 (1.38–1.55)
*Established vascular disease*	6268 (45.48)	20285 (30.78)	2.56 (2.42–2.71)
CHA_2_DS_2_-VASc score			
Mean (±SD)	3.07 (±1.62)	2.69 (±1.57)	*p* < 0.001
*<3*	4903 (35.58)	28666 (43.49)	1 (Ref.)
*3–4*	6447 (46.78)	30038 (45.57)	1.95 (1.83–2.07)
*>4*	2431 (17.64)	7205 (10.93)	3.39 (3.13–3.67)
Current use of			
*Antiplatelet drugs*	3691 (26.78)	10748 (16.31)	2.16 (2.07–2.27)
*Oral anticoagulants*	1068 (7.75)	3594 (5.45)	1.51 (1.41–1.62)
*Beta-Blockers*	2030 (14.73)	5813 (8.82)	1.87 (1.77–1.98)
*Alfa-Blockers*	353 (2.56)	1490 (2.26)	1.17 (1.04–1.32)
*ACEIs*	2913 (21.14)	11478 (17.41)	1.40 (1.33–1.46)
*ARBs*	2430 (17.63)	9945 (15.09)	1.25 (1.19–1.32)
*CCBs*	2140 (15.53)	8070 (12.24)	1.42 (1.34–1.49)
*Diuretics*	2474 (17.95)	8794 (13.34)	1.55 (1.47–1.64)
*Paracetamol*	2317 (16.81)	10823 (16.42)	1.15 (1.08–1.22)
*Metamizole*	679 (4.93)	2517 (3.82)	1.40 (1.28–1.52)
*NSAIDs*	1268 (9.20)	6397 (9.71)	1.00 (0.93–1.07)
*Corticosteroids*	308 (2.23)	1081 (1.64)	1.39 (1.22–1.58)
*Opioids*	699 (5.07)	2680 (4.07)	1.29 (1.18–1.40)
*CaS (with or without Vit D)*	611 (4.43)	2811 (4.26)	1.03 (0.94–1.12)
*Hormone replacement therapy^e^*	14 (0.10)	82 (0.12)	0.81 (0.46–1.44)
*SERM*	28 (0.20)	168 (0.25)	0.76 (0.51–1.14)
*Strontium ranelate*	31 (0.22)	107 (0.16)	1.35 (0.91–2.02)
*Calcitonin*	21 (0.15)	128 (0.19)	0.79 (0.50–1.25)
*Denosumab*	6 (0.04)	34 (0.05)	0.76 (0.32–1.81)
*Teriparatide*	9 (0.07)	41 (0.06)	0.98 (0.48–2.03)
*PPIs*	4488 (32.57)	17442 (26.46)	1.44 (1.38–1.51)
*H*_*2*_ *receptor blockers*	322 (2.34)	1133 (1.72)	1.38 (1.22–1.57)

ACEIs, Angiotensin Converting Enzyme Inhibitors; ARBs, Angiotensin II-Receptor Blockers; BMI, body max index; CaS: calcium supplements; CCBs, Calcium-channel blockers; CI, confidence interval; COPD, chronic obstructive pulmonary disease; NSAIDs, Non-steroidal Anti-inflammatory Drugs; OR, odds ratio; PPIs, Proton-pump inhibitors; SD, standard deviation; SERM, selective estrogen receptor modulators.

^a^ Adjusted only for matching factors (age, sex, and calendar year).

^b^ Recorded as such, and/or when patients were using nitrates.

^c^ Recorded as such, and/or when patients were using glucose-lowering drugs.

^d^ Recorded as such, and/or when patients were using lipid-lowering drugs.

^e^ Including tibolone.

The comorbidity and comedication patterns of oB users relative to non-users were explored among controls. Compared to non-users, current users of oBs were mainly women (86.7% vs 44.8%; *p* < 0.001), older (mean age [ ± SD]:77.6 [ ± 8.8] vs 74.1 [ ± 12.6]); *p* < 0.001), had more visits to their PCPs in the last year (mean number of visits [ ± SD]:18.5 [ ± 15.4] vs 13.7 [ ± 13.3]; *p* < 0.001) and presented a higher prevalence of vascular diseases and risk factors, as well as a higher use of comedication (Supplementary Figure S2).

Among IS cases, 434 out of 13,781 (3.15%) were current users of oBs while among controls there were 1,727 out of 65,909 (2.62%), yielding an AOR of 1.15 (95% CI:1.01–1.30). When cases were separated according to the most probable pathophysiological subtype, there were 251 out of 9,213 (2.72%) current users of OBs among cases of non-cardioembolic IS and 1,094 out of 44,212 (2.47%) among controls, yielding an AOR of 1.03 (95% CI: 0.88–1.21); among 4,568 cardioembolic IS cases, 183 (4.01%) were current users of oBs and 633 among 21,697 controls (2.92%), yielding an AOR of 1.35 (95% CI: 1.10–1.66). Such increased AOR of cardioembolic IS did not disappear upon discontinuation (AOR in past users of 1.39; 95% CI:1.06–1.83; Table 2). A statistically significant trend towards higher AORs with longer durations among current users was observed for cardioembolic IS (AOR≤1year = 1.10; 95% CI:0.82–1.49; AOR>1–3 years = 1.41; 95% CI:1.01–1.97 and AOR>3 years = 1.81; 95% CI:1.25–2.62; *p* for trend = 0.001) (Table 2).

**TABLE 2:** Association of oral bisphosphonates with ischemic stroke by duration, overall and by the most probable pathophysiological subtype (cardioembolic, non-cardioembolic).

Overall	Cases (%) N = 13781	Controls (%) N = 65909	Non-adjusted OR^a^ (95% CI)	Adjusted OR^b^ (95% CI)
Non-users	13127 (95.25)	63335 (96.09)	1 (Ref.)	1 (Ref.)
Current users	434 (3.15)	1727 (2.62)	1.17 (1.05–1.31)	1.15 (1.01–1.30)
Duration ≤1 year	172 (1.25)	811 (1.23)	1.00 (0.85–1.18)	0.96 (0.80–1.15)
Duration >1 year	262 (1.90)	916 (1.39)	1.33 (1.15–1.53)	1.32 (1.12–1.54)
>1–3 years	135 (0.98)	524 (0.80)	1.20 (0.99–1.45)	1.19 (0.97–1.47)
>3 years	127 (0.92)	392 (0.59)	1.49 (1.22–1.83)	1.48 (1.19–1.85)
Past users	220 (1.60)	847 (1.29)	1.20 (1.03–1.40)	1.12 (0.95–1.33)
Time since discontinuation				
>1–3 years	111 (0.81)	459 (0.70)	1.13 (0.91–1.39)	1.08 (0.86–1.35)
>3 years	109 (0.79)	388 (0.59)	1.29 (1.04–1.60)	1.19 (0.94–1.50)
**BY PATHOPHYSIOLOGICAL SUBTYPE**				
**Non-cardioembolic stroke**	**Cases (%) N = 9213**	**Controls (%) N = 44212**	**Non-adjusted OR^a^ (95% CI)**	**Adjusted OR^b^ (95% CI)**
Non-users	8834 (95.89)	42591 (96.33)	1 (Ref.)	1 (Ref.)
Current users	251 (2.72)	1094 (2.47)	1.07 (0.92–1.23)	1.03 (0.88–1.21)
Duration ≤1 year	102 (1.11)	521 (1.18)	0.92 (0.74–1.14)	0.89 (0.71–1.12)
Duration >1 year	149 (1.62)	573 (1.30)	1.20 (1.00–1.44)	1.16 (0.95–1.42)
>1–3 years	76 (0.82)	328 (0.74)	1.07 (0.83–1.38)	1.06 (0.81–1.39)
>3 years	73 (0.79)	245 (0.55)	1.37 (1.05–1.79)	1.29 (0.97–1.72)
Past users	128 (1.39)	527 (1.19)	1.12 (0.92–1.37)	1.04 (0.84–1.29)
Time since discontinuation				
>1–3 years	66 (0.72)	290 (0.66)	1.06 (0.81–1.39)	1.02 (0.77–1.36)
>3 years	62 (0.67)	237 (0.54)	1.21 (0.91–1.60)	1.07 (0.79–1.45)
**Cardioembolic stroke**	**Cases (%) N = 4568**	**Controls (%) N = 21697**	**Non-adjusted OR^a^ (95% CI)**	**Adjusted OR^b^ (95% CI)**
Non-users	4293 (93.98)	20744 (95.61)	1 (Ref.)	1 (Ref.)
Current users	183 (4.01)	633 (2.92)	1.36 (1.14–1.61)	1.35 (1.10–1.66)
Duration ≤1 year	70 (1.53)	290 (1.34)	1.14 (0.88–1.49)	1.10 (0.82–1.49)
Duration >1 year	113 (2.47)	343 (1.58)	1.54 (1.23–1.91)	1.57 (1.21–2.04)
>1–3 years	59 (1.29)	196 (0.90)	1.41 (1.05–1.90)	1.41 (1.01–1.97)
>3 years	54 (1.18)	147 (0.68)	1.70 (1.24–2.34)	1.81 (1.25–2.62)
Past users	92 (2.01)	320 (1.47)	1.33 (1.05–1.68)	1.39 (1.06–1.83)
Time since discontinuation				
>1–3 years	45 (0.99)	169 (0.78)	1.24 (0.89–1.74)	1.36 (0.94–1.96)
>3 years	47 (1.03)	151 (0.70)	1.42 (1.02–1.98)	1.45 (0.99–2.12)

CI, confidence interval; OR, odds ratio.

^a^ Adjusted only for matching factors (age, sex and calendar year).

^b^ Adjusted for matching factors (age, sex and calendar year) plus number of primary care physician (PCP) visits in the last year, smoking, alcohol dependence (recorded as such by PCP), body mass index, transient ischemic attack, ischemic heart disease (including history of acute myocardial infarction or angina pectoris - recorded as such and/or use of nitrates), thromboembolism, heart failure, peripheral artery disease, hypertension, diabetes (recorded as such, and/or use of glucose-lowering drugs), dyslipidemia (registered as such, and/or use of lipid-lowering drugs), hyperuricemia (asymptomatic and gout), chronic obstructive pulmonary disease, rheumatoid arthritis, and chronic renal failure, and use within the last 30 days of antiplatelet agents, beta-blockers, alpha blockers, angiotensin-converting enzyme inhibitors, angiotensin II, receptor antagonists, calcium antagonists, diuretics, paracetamol, metamizole, non-steroidal anti-inflammatory drugs, corticosteroids, opioids, calcium with/without vitamin D supplements, hormonal replacement therapy, estrogen receptor modulators, strontium ranelate, calcitonin, denosumab, teriparatide, proton pump inhibitors and H2-receptor antagonists.

**FIGURE 2:**
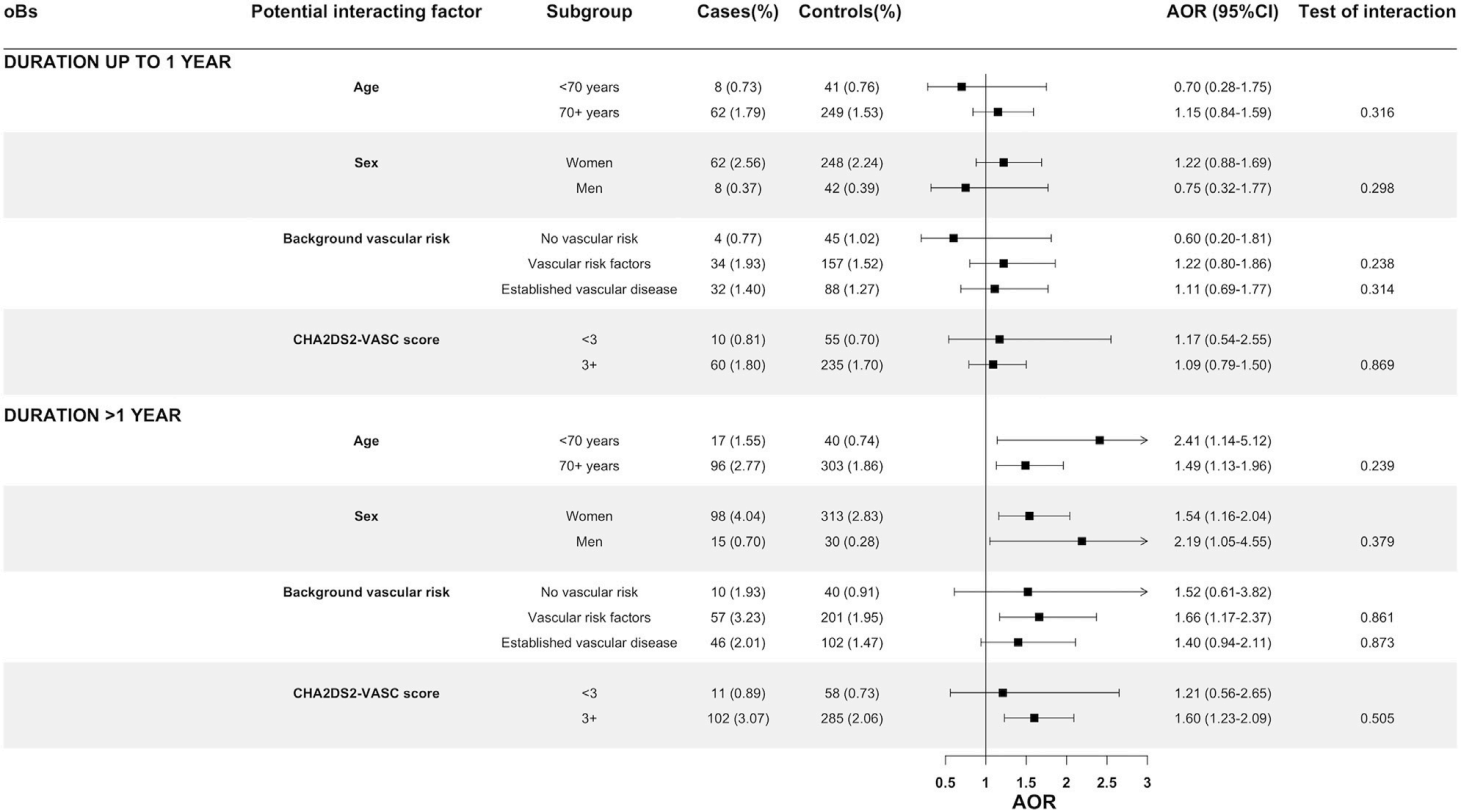
Oral bisphosphonates and its association with cardioembolic ischemic stroke by age, sex, background vascular risk and CHA2DS2-VASc score. Abbreviations: AOR, Adjusted odds ratio; CI, Confidence Interval. Definitions of different categories of vascular risk: (1) established vascular disease, those with a history of ischemic heart disease (AMI or angina pectoris), heart failure, transient ischemic attack, peripheral arterial disease or diabetes; (2) one or more vascular risk factors: those with a history of hypertension, dyslipidemia, chronic renal failure, current smoking, or body mass index >30 kg/m2 (and none of the conditions mentioned in the first point); (3) no known vascular risk factor or disease: the remainder.

Separated by duration (up to 1 year and more than 1 year), no evidence of statistical interaction was found between oB use and age group (<70 years and 70 or over), sex, background vascular risk or CHA2DS2-VASc with respect to the association with either cardioembolic IS (Figure 2) or non-cardioembolic IS (Supplementary Figure S3).

**FIGURE 3:**
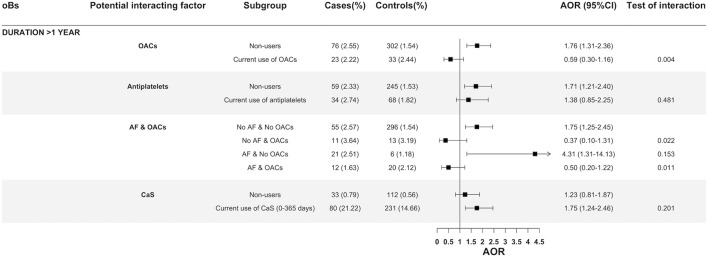
Oral bisphosphonates and its association with cardioembolic ischemic stroke stratified by use of oral anticoagulants, antiplatelets and history of atrial fibrillation with or without oral anticoagulants. Abbreviations: AF, Atrial fibrillation; AOR: Adjusted odds ratio; CI, Confidence Interval; CS, Calcium supplements; OACs, Oral anticoagulants.

The interaction of long-term use of oBs (more than 1 year) with AF and current use of antithrombotic drugs on cardioembolic IS was explored in specific stratified analyses (Figure 3). While an increased AOR associated with the long-term current use of oBs was clearly observed among non-users of OACs (AOR = 1.76; 95% CI:1.31–2.36) and non-users of antiplatelets (AOR = 1.71; 95% CI:1.21–2.40), it completely disappeared in the stratum of current users of OACs (AOR = 0.59; 95% CI:0.30–1.16; test of interaction *p* = 0.004) or partially in the stratum of current users of antiplatelets (AOR = 1.38; 95% CI:0.85–2.25; test of interaction *p* = 0.481) (Figure 3). Among individuals with a recorded AF and no prescriptions of OACs, the long-term use of oBs showed a substantial increase in the AOR of cardioembolic IS (AOR = 4.31; 95%:1.31–14.13) as compared to non-use. No increased AOR associated with the long-term use of oBs was observed in the strata of patients on OACs, regardless they had or not a recorded AF (Figure 3). An interaction with CaS was also suggested, but the statistical significance was not reached (*p* = 0.201) (Figure 3).

**FIGURE 4:**
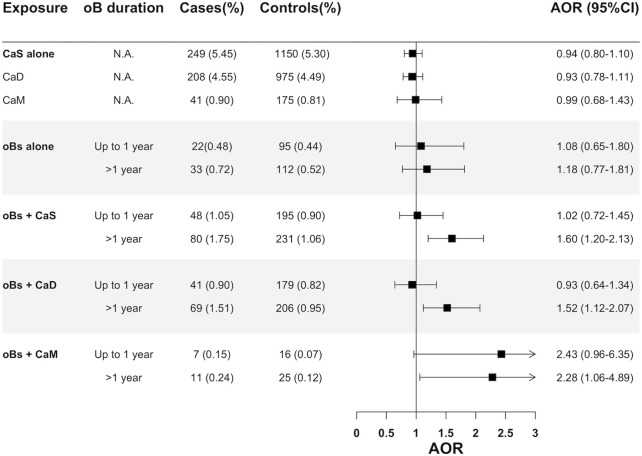
Assessment of the interaction (additive scale) between oral bisphosphonates and calcium supplements on cardioembolic ischemic stroke. Abbreviations: AOR, Adjusted odds ratio; CaD, Calcium supplements with vitamin D; CaM: Calcium supplements without vitamin D; CI, Confidence interval; CS, Calcium supplement; N.A, Not applicable.

We also examined the potential interaction of long-term use of oBs with CaS in the additive scale and found that a statistically significant association with cardioembolic IS was only observed when long-term users of oBs were also users of CaS (AOR>1year, CaS users = 1.60; 95% CI:1.20–2.13), with both CaS with vitamin D (AOR>1year = 1.52; 95% CI:1.12–2.07) and CaS without vitamin D (AOR>1 year, = 2.28; 95% CI:1.06–4.89), while no significant association was observed when long-term oBs were used alone (AOR>1year among CaS nonusers = 1.18; 95% CI:0.77–1.81) (Figure 4).

By active principle, we observed a similar pattern with all oBs, with the exception of risedronic acid, for which we did not find an association (see Table 3).

**TABLE 3:** Association of individual oral bisphosphonates with cardioembolic ischemic stroke and effect of duration.

	Cases (%) N = 4568	Controls (%) N = 21697	Non-adjusted OR^a^ (95% CI)	Adjusted OR^b^ (95% CI)
**Alendronic acid**				
Non-users	4409 (96.52)	21175 (97.59)	1 (Ref.)	1 (Ref.)
Current users	94 (2.06)	331 (1.53)	1.32 (1.04–1.67)	1.31 (1.00–1.71)
Duration ≤1 year	34 (0.74)	161 (0.74)	0.99 (0.68–1.43)	0.96 (0.63–1.44)
Duration >1 year	60 (1.31)	170 (0.78)	1.63 (1.21–2.20)	1.66 (1.18–2.33)
>1–3 years	31 (0.68)	95 (0.44)	1.52 (1.01–2.28)	1.40 (0.90–2.20)
>3 years	29 (0.63)	75 (0.35)	1.78 (1.16–2.75)	2.05 (1.26–3.33)
Past users	65 (1.42)	191 (0.88)	1.56 (1.17–2.08)	1.59 (1.14–2.20)
**Risedronic acid**				
Non-users	4472 (97.90)	21359 (98.44)	1 (Ref.)	1 (Ref.)
Current users	54 (1.18)	192 (0.88)	1.31 (0.97–1.79)	1.08 (0.76–1.53)
Duration ≤1 year	26 (0.57)	94 (0.43)	1.33 (0.86–2.06)	1.08 (0.67–1.75)
Duration >1 year	28 (0.61)	98 (0.45)	1.30 (0.85–1.99)	1.08 (0.67–1.73)
>1–3 years	18 (0.39)	52 (0.24)	1.60 (0.93–2.76)	1.39 (0.76–2.52)
>3 years	10 (0.22)	46 (0.21)	0.97 (0.49–1.94)	0.75 (0.35–1.60)
Past users	42 (0.92)	146 (0.67)	1.32 (0.93–1.86)	1.25 (0.85–1.83)
**Ibandronic acid**				
Non-users	4521 (98.97)	21531 (99.23)	1 (Ref.)	1 (Ref.)
Current users	35 (0.77)	116 (0.53)	1.37 (0.94–2.01)	1.44 (0.95–2.20)
Duration ≤1 year	14 (0.31)	60 (0.28)	1.06 (0.59–1.90)	1.10 (0.58–2.08)
Duration >1 year	21 (0.46)	56 (0.26)	1.71 (1.03–2.83)	1.82 (1.04–3.18)
>1–3 years	13 (0.28)	43 (0.20)	1.39 (0.74–2.59)	1.56 (0.79–3.10)
>3 years	8 (0.18)	13 (0.06)	2.75 (1.13–6.66)	2.54 (0.96–6.75)
Past users	12 (0.26)	50 (0.23)	1.06 (0.56–2.00)	1.33 (0.67–2.64)
**Etidronic acid**				
Non-users	4565 (99.93)	21679 (99.92)	1 (Ref.)	1 (Ref.)
Current users	1 (0.02)	7 (0.03)	0.69 (0.09–5.64)	0.38 (0.04–3.35)
Past users	2 (0.04)	11 (0.05)	0.86 (0.19–3.89)	0.60 (0.11–3.28)

CI, confidence interval; OR, odds ratio.

^a^ Adjusted only for matching factors (age, sex and calendar year).

^b^ Adjusted for matching factors (age, sex and calendar year) plus number of primary care physician (PCP) visits in the last year, smoking, alcohol dependence (recorded as such by PCP), body mass index, transient ischemic attack, ischemic heart disease (including history of acute myocardial infarction or angina pectoris—recorded as such and/or use of nitrates), thromboembolism, heart failure, peripheral artery disease, hypertension, diabetes (recorded as such, and/or use of glucose-lowering drugs), dyslipidemia (registered as such, and/or use of lipid-lowering drugs), hyperuricemia (asymptomatic and gout), chronic obstructive pulmonary disease, rheumatoid arthritis, and chronic renal failure, and use within the last 30 days of antiplatelet agents, beta-blockers, alpha blockers, angiotensin-converting enzyme inhibitors, angiotensin II, receptor antagonists, calcium antagonists, diuretics, paracetamol, metamizole, non-steroidal anti-inflammatory drugs, corticosteroids, opioids, calcium with/without vitamin D supplements, hormonal replacement therapy, estrogen receptor modulators, strontium ranelate, calcitonin, denosumab, teriparatide, proton pump inhibitors and H2-receptor antagonists.

In the sensitivity analyses: 1) Ever users of oBs showed the same pattern observed in the main analysis: a specific positive association with cardioembolic IS and a clear trend with duration (Supplementary Table S3); 2) the inclusion of prevalent users of oBs made the association to vanish overall (AOR = 1.06; 95% CI:0.89–1.25), even in long-term current users (AOR>1–3 years = 1.14; 95% CI:0.89–1.45 and AOR>3 years = 1.16; 95% CI:0.89–1.53) (Supplementary Table S4).

## 4 Discussion

The main findings of this study are as follows: (1) the use of oBs showed a positive association with cardioembolic IS, in a duration-dependent manner, while no association with of non-cardioembolic IS was observed; (2) the positive association with cardioembolic IS remains high for a long time upon discontinuation; (3) no evidence of statistical interaction with sex, age, background vascular risk or CHA2DS2-VASc was observed; (4) the use of OACs completely blunted the positive association of oBs with cardioembolic IS, while the use of antiplatelets did so partially; 5) the association of long-term use of oBs with cardioembolic IS associated with the long-term use of oBs was only observed among users of CaS; and 6) all active principles studied presented the same pattern with the exception of risedronic acid.

The main novelty of the present research is the separated analysis of IS by the most probable pathophysiological subtype. None of the previously published studies reported results this way. Kim et al. published in 2015 a meta-analysis of RCTs that reported cardiovascular events associated with bisphosphonates and found a pooled OR of 0.99 (95% CI: 0.82–1.19) for stroke of any type based on six clinical trials (Kim et al., 2015). Reid et al. published a 6-year clinical trial with zoledronic acid vs placebo in women with osteopenia, not included in such meta-analysis, and reported a rate ratio of stroke of any type of 0.90 (95% CI:0.49–1.66) (Reid et al., 2020). Up to now, eight observational studies have been published aiming to evaluate the relationship between bisphosphonates and IS: four found a risk reduction (Kang et al., 2013; Sing et al., 2018; Gegechkori et al., 2019; Rodríguez et al., 2020), three no association (Christensen et al., 2011; Wang et al., 2016; Asghar et al., 2019) and one an increased risk (Vestergaard et al., 2011). None, however, provided data separated by the main pathophysiological subtype and the majority did not employ a new-user design. Both elements, along with the duration of treatment, proved critical in our study. When, in a sensitivity analysis, we included prevalent users and put all types of IS together we found an AOR of 0.97 (95% CI: 0.88–1.07), which would have led us to conclude that there were no association between bisphosphonates and IS. These results emphasize the importance of an appropriate definition of the outcome and the causal model, as well as the application of the new-user design in pharmacoepidemiology.

Several mechanisms have been postulated to explain the association of bisphosphonates with AF, including fibrosis and myocardial remodeling, calcium handling abnormalities and inflammatory changes (Fazmin et al., 2020), and these mechanisms may also be argued to explain the increased risk of cardioembolic IS. However, our results suggest that oBs are associated with an increase in the risk of cardioembolic IS only after long-term exposure which means that the underlying biological substrate should only develop at long run. In that sense, arrhythmogenesis associated with short-term abnormalities of electrolyte handling or acute inflammatory changes induced by bisphosphonates, as reported by several experimental studies (Pazianas et al., 2010), might not be compatible with our results. It seems more plausible the idea of a progressive atrial fibrosis and cardiac remodeling, possibly as a consequence of the antiangiogenic activity of bisphosphonates (Pazianas et al., 2010). In the same vein, several authors have suggested that the mechanism involved in the osteonecrosis of the jaw and atypical femoral fractures induced by bisphosphonates could also occur as a consequence of this vascular insufficiency (Kuroshima et al., 2019; Lockwood et al., 2019) and both seem to be duration-dependent phenomena. The hypothesis of an atrial fibrosis and cardiac remodeling fits well with the model proposed by Kamel et al. (2016) for cardioembolic IS. These authors propose that an abnormal atrial substrate (an atrial cardiopathy), would be the main underlying disorder leading to a thrombogenic state and subsequent cardiac embolism without the need of concurring a rhythm disorder, although AF is, obviously, an important marker of atrial cardiopathy and may increase by itself the risk of cardiac embolism. This model would explain the association of oBs with cardioembolic IS we observed in patients without a record of AF, though an undetected AF may also be a plausible explanation (see Supplementary Figure S1). The finding that risedronic acid does not share the pattern of other bisphosphonates is intriguing and should be further explored. This bisphosphonate is less positively charged on their nitrogen atom in the R2 group than the others (Russell et al., 2008) and this could lead to a less cytosolic penetration and less extra-skeletal effects (Pazianas et al., 2010). The observation that long-term oBs was associated with a significant cardioembolic IS only among users of CaS suggests that calcium may be playing a crucial mechanistic role. It is conceivable that an alteration of calcium dynamics in atrial cardiomyocytes induced by bisphosphonates, as reported (Kemeny-Suss et al., 2009) may be further impaired if there is a calcium overload, perhaps leading to cell damage (Oe et al., 1994) and, ultimately, inducing an atrial cardiopathy that can promote cardiac embolism. The action of calcium on the coagulation system may also be playing a role (Bristow et al., 2015). Regarding the potential role of the interaction between oBs and CaS, it is interesting to note that in the randomized clinical trial with zoledronic acid in women with osteopenia carried out by Reid et al. (2018, 2020), no increased risk of stroke was observed, and the percentage of women using calcium supplements was as low as 2%.

According to data from The Global Burden of Diseases, Injuries, and Risk Factors Study (GBD, 2019 Stroke Collaborators, 2021), the annual standardized incidence of IS was estimated in 94.51 cases per 100,000 persons. In our study, the proportion of cardioembolic IS was around 33%. Applying this proportion to the global standardized incidence we may estimate the annual incidence of cardioembolic IS in 31 per 100,000 persons (resulting in a cumulative incidence of around 155 per 100.000 persons for 5 years). Then, assuming that the use of bisphosphonates for 5 years produces an increased risk of 81% (the AOR found for a duration longer than 3 years in our study), the number of patients to be treated over this period of time to obtain a case of cardioembolic IS attributable to oBs (the NNH), would be 796 patients (Supplementary Table S5), much larger than the number needed to treat (NNT) estimated in the FIT trial for alendronic acid to prevent a hip fracture over 5 years (NNT = 66) (Black et al., 2000), which suggests that the benefit-risk balance of bisphosphonates remains clearly favorable.

The following strengths of our study should be highlighted: 1) although the access to the data by the investigators was retrospective, the PCPs collected the clinical information prospectively; 2) PCPs fill the prescriptions using the computer system making misclassification of the exposure highly unlikely; 3) controls were randomly drawn from the underlying cohort following a incidence-density sampling, which prevents from a control-selection bias (Rothman et al., 2013) and 4) only new users of bisphosphonates were considered to avoid a prevalent-user bias. Among limitations: 1) the present study is observational in nature and a residual confounding due to unmeasured or unknown confounders is still possible; in this sense, although we included chronic renal failure in the adjusted models, we did not have accurate data on the evolution of glomerular filtration rate, which may be a factor that could modify the effect of oral bisphosphonates at long-term; 2) treatment adherence, as in any clinical study, is not guaranteed; 3) despite the validation effort to determine the most probable pathophysiological subtype of IS (cardioembolic and non-cardioembolic) there may still be some misclassification; and 4) zoledronic acid was not included in the study and thus results cannot be extrapolated to this drug, although the evidence of an association with AF is particularly great with this bisphosphonate.

These results need to be confirmed through other pharmacoepidemiological studies, as the scientific logic demands, especially using a longitudinal design. Also, clinical studies designed to examine whether the long-term use of bisphosphonates is associated or not with an atrial cardiopathy detected by a standard ECG or echocardiogram (Kamel et al., 2016) would be informative and are encouraged. Meanwhile, clinicians should be vigilant to detect an AF in patients long-term treated with bisphosphonates and consider anticoagulation if detected. Unfortunately, the discontinuation of bisphosphonates may not be a helpful risk minimization measure, as a residual long-term effect seems to persist (consistent with their long biological half-life). The observation that the greatest effect concentrates among users of oBs who concomitantly used CaS adds evidence against the routine use of CaS in association with oBs in osteoporosis (Reid et al., 2015). Further studies are needed to clarify the underlying mechanisms and whether oBs without CaS are completely devoid of such a risk.

In conclusion, the results of the present study are compatible with the hypothesis that the long-term use of bisphosphonates is specifically associated with an increased risk of cardioembolic IS, which seems especially high in patients with a recorded AF not treated with anticoagulants. Nonetheless, in patients without a recorded AF, we also found an association between the use of oBs and cardioembolic IS, which may be explained by an undetected AF and/or an abnormal atrial substrate without arrythmia. The use of anticoagulants seems to be an effective protective measure.

## Data Availability

Anonymized data not published within this article will be made available by reasonable request from any qualified investigator, provided that the owner of BIFAP (the AEMPS) authorized specifically the data transfer.
