# Supplementary material for: Association of oral bisphosphonates with cardioembolic ischemic stroke: a nested case-control study
Source: Front Pharmacol. 2023 May 26;14:1197238. doi: 10.3389/fphar.2023.1197238 (PMC10250719; doi:10.3389/fphar.2023.1197238)
Supplement: Supplementary file 1 [file DataSheet1.pdf]

frontiers

Supplementary Material

RISK OF CARDIOEMBOLIC ISCHAEMIC STROKE AMONG NEW USERS OF ORAL BISPHOSPHONATES: A NESTED CASE-CONTROL STUDY

Sara Rodríguez-Martín[1†], Diana Barreira-Hernández[1†], Ramón Mazzucchelli[2], Miguel Gil[3], Alberto Garcia-Lledó[4,5], Laura Izquierdo-Esteban[6], Ana Pérez-Gómez[5,7], Antonio Rodríguez-Miguel[1], Francisco J. de Abajo[1,8*]

[1]Department of Biomedical Sciences (Pharmacology), University of Alcalá (IRYCIS), Alcalá de Henares, Spain

[2]Rheumatology Department, University Hospital "Fundación Alcorcón", Alcorcón, Spain

[3]Division of Pharmacoepidemiology and Pharmacovigilance, Spanish Agency on Medicines and Medical Devices (AEMPS, by its Spanish acronym), Madrid, Spain

[4]Department of Cardiology, University Hospital "Príncipe de Asturias", Alcalá de Henares, Spain

[5]Department of Medicine, University of Alcalá, Alcalá de Henares, Spain

[6]Department of Neurology, Stroke Unit, University Hospital "Príncipe de Asturias", Alcalá de Henares, Spain

[7]Department of Rheumatology, University Hospital "Príncipe de Asturias", Alcalá de Henares, Spain

[8]Clinical Pharmacology Unit, Universty Hospital "Príncipe de Asturias", Alcalá de Henares, Spain

[†]These authors contributed equally to this work and share first authorship

[*] Correspondence:
Prof. Francisco J. de Abajo
Departamento de Ciencias Biomédicas
Universidad de Alcalá
Carretera Madrid-Barcelona, km 33.5
28805 Alcalá de Henares. Madrid, Spain
Tel. +34 918852593
Fax: +34 918854591
Email address: francisco.abajo@uah.es

1 Supplementary Methods

1.1 Data source

BIFAP (Base de datos para la Investigación Farmacoepidemiológica en el Ámbito Público) is a primary care database managed by the Spanish Agency of Medicines and Medical Devices (AEMPS) that contains pseudonymized information on clinical diagnoses, medical comments in free text, laboratory tests, vaccinations, complementary explorations and prescriptions, prospectively recorded by primary care physicians (PCPs) in the National Health System. Clinical events are recorded using the International Classification of Primary Care, version 2 (ICPC-2) or the International Classification of Diseases, version 9, Clinical Modification (ICD-9-CM), depending on the region. All prescriptions written by the PCPs are recorded including product name, quantity, dosing regimens, indication and date of prescription. The BIFAP population is representative of the population receiving healthcare in Spain (Maciá-Martínez et al., 2020). The information in the database is enriched with free text annotations of the PCPs. BIFAP has been extensively validated through multiple pharmacoepidemiological studies in different areas, including cardiovascular (Rodríguez-martín et a., 2020) To conduct this study, the 2016 version was used, which contains data from 7.6 million patients, with an average of 5.1 years of follow-up (38.8 million person-years), from nine different Spanish regions. The study period encompasses 14 years, from January 1, 2002 to December 31, 2015.

1.2 Stroke case validation procedures

BIFAP contains information on clinical diagnoses that PCPs record as part of their routine clinical practice. In order to adequately perform pharmacoepidemiological studies in BIFAP, the event of interest recorded in the data source is validated.

In BIFAP database, predefined case-finding algorithms (CFA) for clinical events are available based on proper code selection within ICPC-BIFAP or ICD-9 codes. The methodology to normalize the diagnosis information in BIFAP is detailed elsewhere24.

The validation procedures of the predefined stroke CFA included the following steps:

1. Definition of the study population and identification of potential stroke cases retrieved by the stroke CFA.
2. Manual review of the patient's electronic healthcare records -including clinical notes-, in a random sample of 1000 potential cases, to confirm or rule out the diagnosis according pre-specified criteria. This review also included the validity of the date of the event.
3. Estimation of the Positive Predictive Value (PPV) of the predefined stroke CFA to identify valid incident ischemic strokes.
4. Stroke CFA refinement strategies: Using natural language processing (NLP) techniques to increase the PPV in those subsets with PPV lower than 80%.

1.2.1 Validation of the predefined stroke CFA in BIFAP

Predefined CFA for ischemic stroke, hemorrhagic stroke or unspecified stroke are available in BIFAP database. After excluding hemorrhagic strokes, we identified through the CFA 24094 ischemic and 15868 unspecified strokes identified, totaling 39962 cases.

Of them, a sample of 1000 cases was randomly selected for validation purposes. The electronic healthcare records (EHR) –including clinical notes- of the sample were manually reviewed, blinded to drug exposure, independently by two investigators (SRM and DBH) looking for additional information to confirm that it was a true case of incident stroke. Discrepancies were settled by the entire research group. According to this, patients were classified according to pre-specified criteria as:

- Valid cases: when additional evidence was found in the EHRs that confirmed the diagnosis;
- Cases with insufficient information: when there was no additional information in the EHRs to support or rule-out the stroke diagnosis;
- Non-case: when the available information allowed to rule out the diagnosis of incident stroke.

The review of the random sample of 1000 potential stroke cases showed the following results: 641 valid cases; 168 insufficient information and 191 non-cases.

1.2.2 Refinement procedures to increase the PPV of the predefined ischemic stroke CFA.

Natural language processing techniques were implemented, in order to identify:

a) Semantic patterns in clinical notes with high likelihood to be in the clinical notes of the valid stroke cases.
b) Semantic patterns in clinical notes with high likelihood to be in the clinical notes of the non-cases and low likelihood to be in the clinical notes of valid cases.

Then, semantic patterns identified in (a) were added to the pre-defined stroke CFA as additional criteria for stroke case selection and semantic patterns identified in (b) were used to consider them as non-cases.

Using this refinement, the resulting PPV in the random sample increased to 87,1% and consequently, the refined incident stroke CFA was applied to all potential stroke cases. A total number of 14374 cases was included in the case-control study.

1.2.3 Identification of the main pathophysiological subtype among ischemic stroke cases

In this step, the free text associated with the diagnosis was examined. The validation strategy focused on identifying and exclude from the study stroke cases of probable cardioembolic origin, as well as other rare causes (e.g. vasculitis, vascular dissection, drug abuse, etc,).

The following main criteria were applied to identify cardioembolic stroke cases:

- Cardioembolic text criterion: the word "cardioembolic "or other related terms was sought in text comments associated with the stroke diagnosis.
- Atrial fibrillation criterion: patients with a diagnosis of atrial fibrillation prior to the stroke event or within three months of stroke diagnosis were identified.
- Oral anticoagulant use criterion: patients on oral anticoagulant therapy at the time of stroke diagnosis (or up to three months prior to diagnosis) or starting treatment in a three-month window since stroke diagnosis.

The following additional criteria were used when at least one of the above was met:

- Antiarrhythmic use criterion: patients on treatment with antiarrhythmic drugs (class IC and III) at the time of stroke diagnosis (or up to three months prior to diagnosis) or starting treatment in a three-month window since stroke diagnosis. In order to consider a case as cardioembolic stroke, it was required to have some other of the aforementioned criteria in addition to the use of antiarrhythmic drugs.
- Mitral valve prosthesis/stenosis criterion: patients with a record of mechanical valve prosthesis or mitral stenosis prior to stroke diagnosis or up to three months after the event. In order to consider a case as cardioembolic, it was required to have some other of the aforementioned criteria in addition to the record of mitral valve prosthesis or stenosis.

Regardless the criteria listed above, all patients who had text strings in their commentaries associated with the stroke diagnosis compatible with the words atherothrombotic, thrombogenic, or lacunar (including related terms) were classified as non-cardioembolic stroke.

Patients with a free-text describing a vascular dissection, cocaine abuse and vasculitis as the probable cause of the stroke were identified in a distinct category and excluded from most analyses.

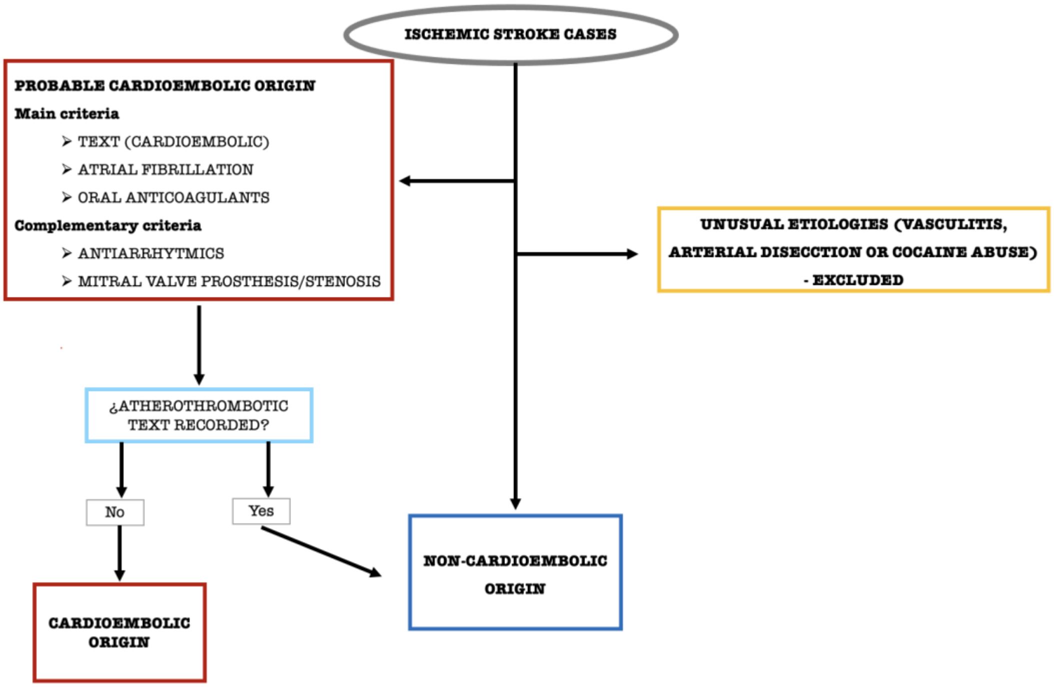

Ischemic stroke cases classified as probable cardioembolic origin according to the criteria applied.

Criteria	Number of cases			
	As a sole criterion	*Combined with any other (non-mutually exclusive)*	*Combined with any other criteria shown below, in a hierarchical order (mutually exclusive)*	*Total (mutually exclusive)*
**Cardioembolic text**	98	1431	1431	1529
**Atrial fibrillation[a]**	478	3080[c]	1944[c]	2422
**OAC use [a,b]**	661	3192[d]	168[d]	829
**Valve prosthesis/mitral stenosis[a]**	0	355[e]	0	0
**Anti-arrhythmics[a]**	0	1366[f]	0	0
**Subtotal**	1237 (25.9%)	-	3543 (74.1%)	4780 (100%)
**Total**				4780 (100%)

[a]Recorded either before the index date or within three months after the event

[b]OAC: Oral anticoagulants

[c] It means that 3080 cases had a record of atrial fibrillation plus at least one other criterion and that 1944 cases had atrial fibrillation plus at least one other criteria different from cardioembolic text

[d] It means that 3192 cases had a record of an OAC prescription plus at least one other criterion and that 168 cases had an OAC prescription plus at least one other criteria different from cardioembolic text and atrial fibrillation

[e] It means that all 355 cases had a record of valve prosthesis/mitral stenosis plus at least any of the criteria listed above (cardioembolic text, atrial fibrillation and/OAC prescription)

[f] It means that all 1366 cases had a record of antiarrhythmic prescriptions plus at least any of the criteria listed above (cardioembolic text, atrial fibrillation and/OAC prescription).

2 Supplementary Figures and Tables

2.1 Supplementary Figures

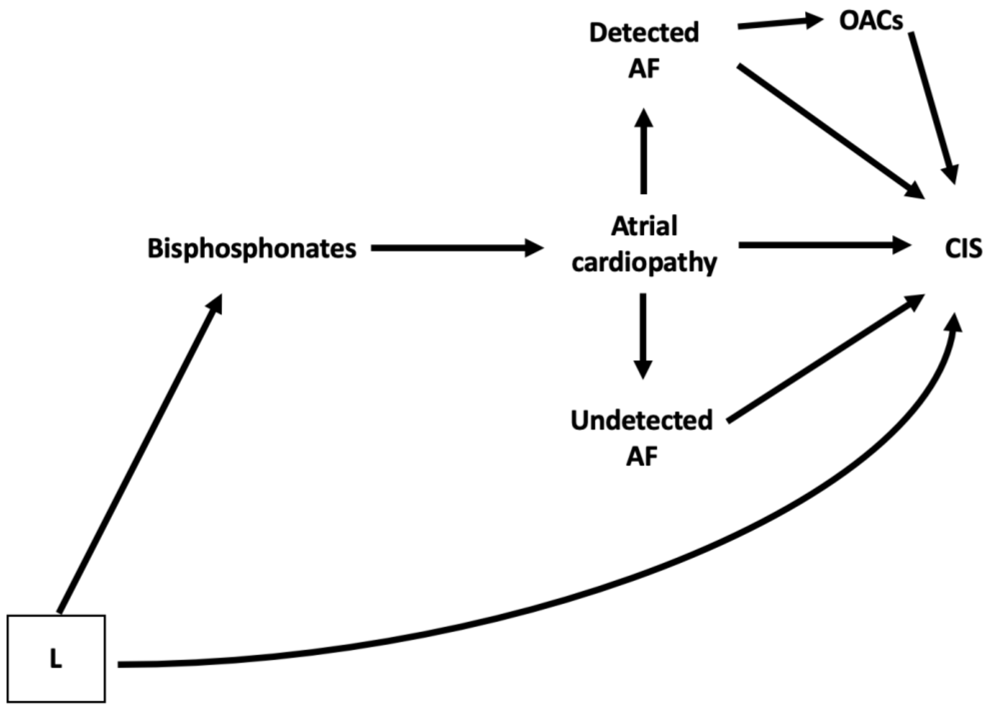

Supplementary Figure 1. Postulated causal diagram.

The arrows connecting bisphosphonates with Atrial cardiopathy and AF (clinical and subclinical) and AF with CIS represent the postulated causal pathway. The possibility that AF can be treated with OACs, a strong protective factor of CIS, is also reflected in the causal graph. The square surrounding L, denotes that the effect of bisphosphonates on CIS is conditioned (or adjusted) on such variables.

Abbreviations: L: Vector of potential confounders; AF: Atrial fibrillation; OACs: Oral anticoagulants; CIS: cardioembolic ischemic stroke.

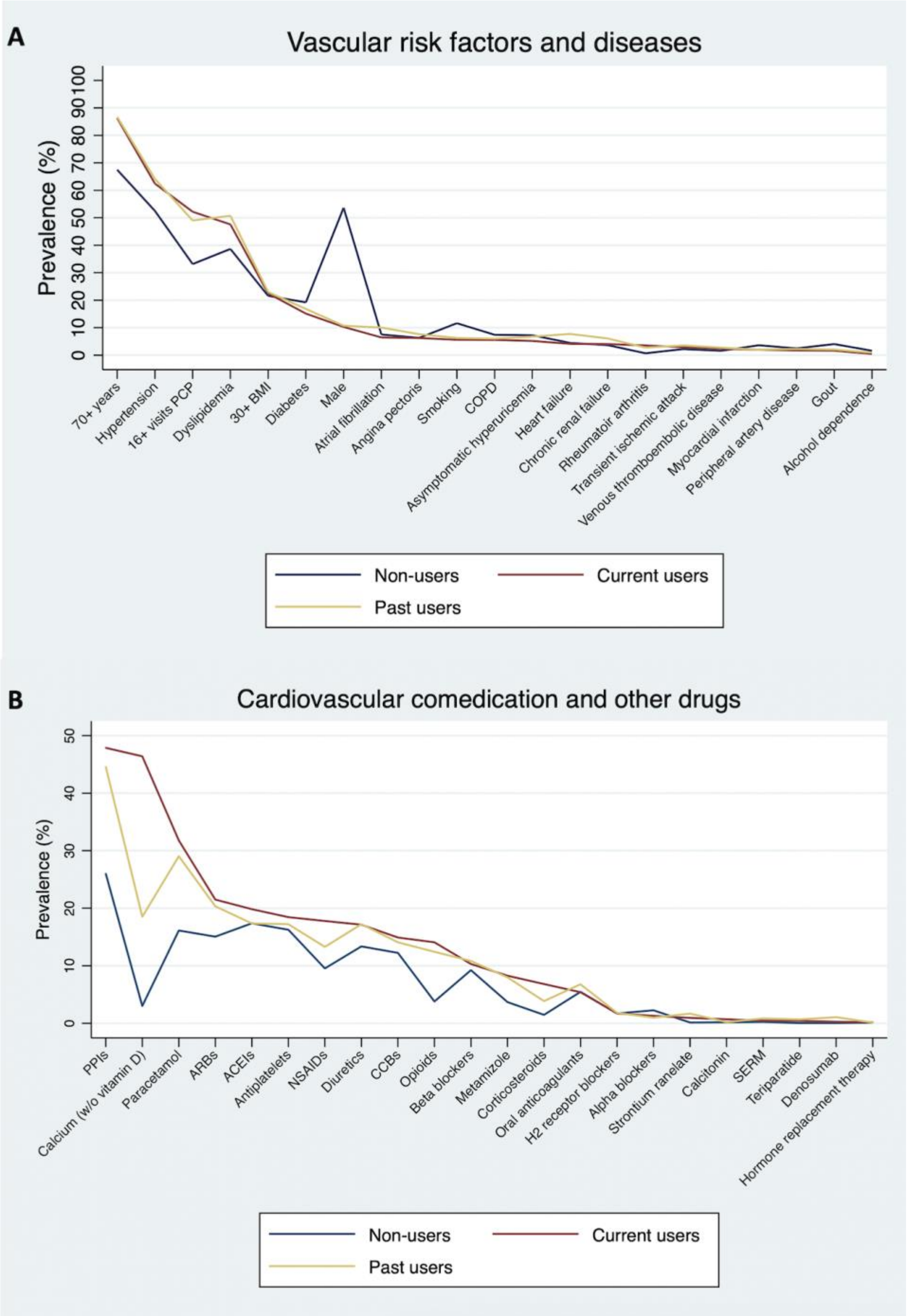

Supplementary Figure 2. Distribution of vascular risk factors or diseases (A) and relevant comedication (B) in oral bisphosphonates current users as compared to non-users and past users among controls (at index date).

Abbreviations: ACEIs: Angiotensin Converting Enzyme Inhibitors; ARBs: Angiotensin II-Receptor Blockers; BMI: Body Max Index; CaS: Calcium supplements; CCBs: Calcium-channel blockers; COPD: chronic obstructive pulmonary disease; NSAIDs: Non-steroidal Anti-inflammatory Drugs; PPIs: Proton-pump inhibitors; SERM: Selective estrogen receptor modulators; w/o: with or without.

- Angina pectoris: recorded as such, and/or when patients were using nitrates.
- Diabetes: recorded as such, and/or when patients were using glucose-lowering drugs.
- Dyslipidemia: recorded as such, and/or when patients were using lipid-lowering drugs.
- Hormone replacement therapy: including tibolone.

oBs	Potential interacting factor	Subgroup	Cases(%)	Controls(%)	AOR (95%CI)	Test of interaction
**DURATION UP TO 1 YEAR**						
	Age	<70 years	23 (0.70)	113 (0.70)	1.05 (0.62-1.78)	
		70+ years	79 (1.33)	408 (1.45)	0.87 (0.67-1.13)	0.531
	Sex	Women	87 (2.05)	426 (2.18)	0.95 (0.74-1.23)	
		Men	15 (0.30)	95 (0.38)	0.62 (0.35-1.12)	0.188
	Background vascular risk	No vascular risk	7 (0.50)	99 (0.94)	0.46 (0.20-1.03)	
		Vascular risk factors	46 (1.20)	273 (1.34)	0.81 (0.58-1.14)	0.211
		Established vascular disease	49 (1.23)	149 (1.12)	1.13 (0.80-1.62)	0.048
	CHA2DS2-VASC score	<3	16 (0.44)	135 (0.65)	0.70 (0.40-1.23)	
		3+	86 (1.55)	386 (1.65)	0.92 (0.71-1.84)	0.467
**DURATION >1 YEAR**						
	Age	<70 years	39 (1.10)	106 (0.66)	1.69 (1.08-2.65)	
		70+ years	113 (1.90)	467 (1.66)	1.05 (0.83-1.32)	0.065
	Sex	Women	128 (3.02)	511 (2.62)	1.16 (0.93-1.44)	
		Men	21 (0.42)	62 (0.25)	1.31 (0.76-2.27)	0.686
	Background vascular risk	No vascular risk	13 (0.93)	88 (0.84)	0.91 (0.47-1.76)	
		Vascular risk factors	75 (1.95)	327 (1.61)	1.06 (0.80-1.41)	0.677
		Established vascular disease	61 (1.53)	158 (1.19)	1.31 (0.94-1.83)	0.334
	CHA2DS2-VASC score	<3	36 (0.98)	124 (0.60)	1.65 (1.08-2.53)	
		3+	113 (2.04)	449 (1.92)	1.03 (0.82-1.30)	0.056

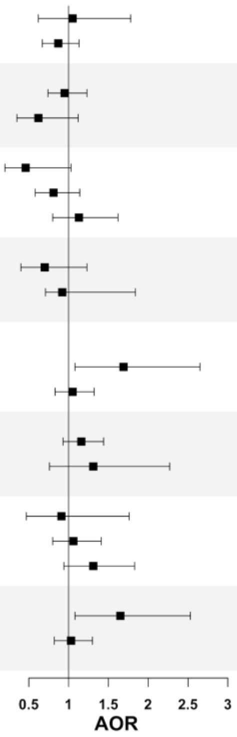

Supplementary Figure 3. Oral bisphosphonates and risk of non-cardioembolic ischemic stroke by age groups, sex, background vascular risk and CHA_2DS_2-VASC score.

Abbreviations: AOR: Adjusted odds ratio; CI: Confidence Interval.
Definitions of different categories of vascular risk: (1) established vascular disease: those with a history of ischemic heart disease (AMI or angina pectoris), heart failure, transient ischemic attack, peripheral arterial disease or diabetes; (2) one or more vascular risk factors: those with a history of hypertension, dyslipidemia, chronic renal failure, current smoking, or body mass index >30 kg/m2 (and none of the conditions mentioned in the first point); (3) no known vascular risk factor or disease: the remainder.

2.2 Supplementary Tables

Supplementary Table 1. Cardioembolic ischemic stroke cases and controls characteristics.

	**Cases (%) N=4568**	**Controls (%) N=21697**	**Non-adjusted OR* (95% CI)**
Age (years); mean (±SD)	76.6 (±11.3)	76.4 (±11.5)	-
Men	2145 (46.96)	10639 (49.03)	-
Visits (last 12 months)			
Up to 5	598 (13.09)	5583 (25.73)	1 (Ref.)
6-15	1507 (32.99)	8113 (37.39)	1.84 (1.66-2.05)
16-24	1118 (24.47)	4281 (19.73)	2.76 (2.47-3.09)
25+	1345 (29.44)	3720 (17.15)	4.02 (3.59-4.51)
Smoking			
Never smoking	1704 (37.30)	7556 (34.83)	1 (Ref.)
Current smoker	540 (11.82)	2230 (10.28)	1.15 (1.03-1.29)
Past smoker	367 (8.03)	1187 (5.47)	1.47 (1.29-1.68)
Unknown	1957 (42.84)	10724 (49.43)	0.82 (0.76-0.88)
Alcohol dependence	112 (2.45)	316 (1.46)	1.78 (1.42-2.22)
BMI kg/m^2			
Up to 24.9	670 (14.67)	3060 (14.10)	1 (Ref.)
25-29	1405 (30.76)	6443 (29.70)	1.01 (0.91-1.12)
30-34	871 (19.07)	3693 (17.02)	1.09 (0.97-1.22)
35-39	231 (5.06)	890 (4.10)	1.19 (1.00-1.41)
40+	87 (1.90)	232 (1.07)	1.72 (1.32-2.23)
Unknown	1304 (28.55)	7379 (34.01)	0.81 (0.73-0.90)
Transient ischemic attack	304 (6.65)	527 (2.43)	2.88 (2.48-3.33)
Ischemic heart disease			
Acute myocardial infarction	391 (8.56)	786 (3.62)	2.81 (2.47-3.20)
Angina pectoris †	545 (11.93)	1445 (6.66)	2.06 (1.85-2.29)
Venous thromboembolic disease	145 (3.17)	381 (1.76)	1.83 (1.51-2.22)
Heart failure	631 (13.81)	1108 (5.11)	3.09 (2.77-3.43)
Atrial fibrillation	1962 (42.95)	1821 (8.39)	9.34 (8.57-10.18)
Peripheral artery disease	249 (5.45)	568 (2.62)	2.23 (1.91-2.61)
Hypertension	3015 (66.00)	12178 (56.13)	1.55 (1.44-1.66)
Diabetes §	1187 (25.99)	4291 (19.78)	1.42 (1.32-1.54)
Dyslipidemia ‖	2126 (46.54)	8734 (40.25)	1.30 (1.21-1.39)
Hyperuricemia			
Asymptomatic	442 (9.68)	1622 (7.48)	1.37 (1.23-1.54)
Gout	263 (5.76)	864 (3.98)	1.58 (1.37-1.82)
COPD	424 (9.28)	1721 (7.93)	1.23 (1.10-1.38)
Rheumatoid arthritis	36 (0.79)	179 (0.82)	0.94 (0.65-1.34)
Chronic renal failure	317 (6.94)	899 (4.14)	1.74 (1.52-1.99)
Background vascular risk			
No risk factors / diseases	519 (11.36)	4411 (20.33)	1 (Ref.)
Risk factors only	1764 (38.62)	10333 (47.62)	1.48 (1.33-1.65)
Established vascular disease	2285 (50.02)	6953 (32.05)	2.96 (2.66-3.30)
CHA_2DS_2-VASc score			
Mean (±SD)	3.40 (±1.61)	2.94 (±1.49)	p<0.001
<3	1241 (27.17)	7892 (36.37)	1 (Ref.)
3-4	2271 (49.72)	11065 (51.00)	2.21 (1.98-2.47)
>4	1056 (23.12)	2740 (12.63)	4.70 (4.10-5.38)

Current use of			
Antiplatelet drugs	1240 (27.15)	3727 (17.18)	2.23 (2.06-2.41)
Oral anticoagulants	1037 (22.70)	1352 (6.23)	5.45 (4.96-6.00)
Beta-Blockers	1076 (23.56)	1976 (9.11)	3.48 (3.19-3.79)
Alfa-Blockers	120 (2.63)	530 (2.44)	1.12 (0.91-1.37)
ACEIs	1066 (23.34)	3907 (18.01)	1.58 (1.46-1.71)
ARBs	877 (19.20)	3572 (16.46)	1.28 (1.17-1.39)
CCBs	771 (16.88)	2855 (13.16)	1.50 (1.38-1.65)
Diuretics	1239 (27.12)	3198 (14.74)	2.55 (2.35-2.77)
Paracetamol	855 (18.72)	3930 (18.11)	1.27 (1.15-1.40)
Metamizole	247 (5.41)	927 (4.27)	1.37 (1.18-1.59)
NSAIDs	359 (7.86)	2200 (10.14)	0.80 (0.70-0.90)
Corticosteroids	120 (2.63)	366 (1.69)	1.60 (1.30-1.98)
Opioids	258 (5.65)	1003 (4.62)	1.27 (1.10-1.46)
CaS (with or without Vit D)	233 (5.10)	1023 (4.71)	1.07 (0.93-1.25)
Hormone replacement therapy ‡	4 (0.09)	15 (0.07)	1.27 (0.41-3.88)
SERM	8 (0.18)	63 (0.29)	0.58 (0.28-1.22)
Strontium ranelate	13 (0.28)	50 (0.23)	1.22 (0.66-2.25)
Calcitonin	8 (0.18)	39 (0.18)	0.99 (0.46-2.13)
Denosumab	3 (0.07)	13 (0.06)	1.01 (0.29-3.57)
Teriparatide	3 (0.07)	17 (0.08)	0.79 (0.23-2.70)
PPIs	1693 (37.06)	6242 (28.77)	1.62 (1.50-1.76)
H_2 receptor blockers	96 (2.10)	397 (1.83)	1.17 (0.93-1.46)

Abbreviations: ACEIs: Angiotensin Converting Enzyme Inhibitors; ARBs: Angiotensin II-Receptor Blockers; BMI: Body Max Index; CaS: Calcium supplements; CCBs: Calcium-channel blockers; CI: Confidence Interval; COPD: chronic obstructive pulmonary disease; NSAIDs: Non-steroidal Anti-inflammatory Drugs; OR: Odds ratio; PPIs: Proton-pump inhibitors; SD: Standard Deviation; SERM: Selective estrogen receptor modulators.

[*] Adjusted only for matching factors (age, sex, and calendar year).
[†] Recorded as such, and/or when patients were using nitrates.
[§] Recorded as such, and/or when patients were using glucose-lowering drugs.
[⁋] Recorded as such, and/or when patients were using lipid-lowering drugs.
[‡] Including tibolone.

Supplementary Table 2. Non-cardioembolic ischemic stroke cases and controls characteristics.

	Cases (%) N=9213	Controls (%) N=44212	Non-adjusted OR* (95% CI)
Age (years); mean (±SD)	73.4 (±12.8)	73.1 (±12.9)	-
Men	4977 (54.02)	24696 (55.86)	-
Visits (last 12 months)			
Up to 5	1853 (20.11)	13408 (30.33)	1 (Ref.)
6-15	3608 (39.16)	16851 (38.11)	1.61 (1.51-1.71)
16-24	2090 (22.69)	7736 (17.50)	2.10 (1.95-2.25)
25+	1662 (18.04)	6217 (14.06)	2.13 (1.97-2.30)
Smoking			
Never smoking	2802 (30.41)	13762 (31.13)	1 (Ref.)
Current smoker	1698 (18.43)	5485 (12.41)	1.69 (1.57-1.82)
Past smoker	583 (6.33)	2450 (5.54)	1.27 (1.15-1.41)
Unknown	4130 (44.83)	22515 (50.93)	0.92 (0.87-0.97)
Alcohol dependence	300 (3.26)	735 (1.66)	2.07 (1.81-2.38)
BMI kg/m^2			
Up to 24.9	1248 (13.55)	5853 (13.24)	1 (Ref.)
25-29	2678 (29.07)	12740 (28.82)	0.99 (0.92-1.07)
30-34	1587 (17.23)	7235 (16.36)	1.03 (0.95-1.12)
35-39	493 (5.35)	1913 (4.33)	1.20 (1.07-1.35)
40+	141 (1.53)	494 (1.12)	1.33 (1.09-1.62)
Unknown	3066 (33.28)	15977 (36.14)	0.91 (0.84-0.98)
Transient ischemic attack	461 (5.00)	939 (2.12)	2.44 (2.18-2.74)
Ischemic heart disease			
Acute myocardial infarction	463 (5.03)	1597 (3.61)	1.46 (1.32-1.63)
Angina pectoris †	694 (7.53)	2716 (6.14)	1.28 (1.17-1.39)
Venous thromboembolic disease	155 (1.68)	697 (1.58)	1.06 (0.89-1.26)
Heart failure	395 (4.29)	1838 (4.16)	1.02 (0.91-1.14)
Atrial fibrillation	50 (0.54)	3119 (7.05)	0.07 (0.05-0.09)
Peripheral artery disease	436 (4.73)	1084 (2.45)	2.02 (1.80-2.26)
Hypertension	5584 (60.61)	22454 (50.79)	1.54 (1.46-1.61)
Diabetes §	2785 (30.23)	8308 (18.79)	1.89 (1.80-1.99)
Dyslipidemia ‖	3957 (42.95)	16937 (38.31)	1.22 (1.16-1.27)
Hyperuricemia			
Asymptomatic	650 (7.06)	3162 (7.15)	1.00 (0.92-1.09)
Gout	434 (4.71)	1782 (4.03)	1.20 (1.08-1.34)
COPD	772 (8.38)	3225 (7.29)	1.19 (1.10-1.30)
Rheumatoid arthritis	63 (0.68)	314 (0.71)	0.95 (0.72-1.24)
Chronic renal failure	470 (5.10)	1568 (3.55)	1.46 (1.31-1.63)
Background vascular risk			
No risk factors / diseases	1391 (15.10)	10535 (23.83)	1 (Ref.)
Risk factors only	3839 (41.67)	20345 (46.02)	1.46 (1.37-1.57)
Established vascular disease	3983 (43.23)	13332 (30.15)	2.39 (2.23-2.56)
CHA_2DS_2-VASc score			
Mean (±SD)	2.90 (±1.60)	2.56 (±1.59)	p<0.001
<3	3662 (39.75)	20774 (46.99)	1 (Ref.)
3-4	4176 (45.33)	18973 (42.91)	1.83 (1.70-1.98)
>4	1375 (14.92)	4465 (10.10)	2.81 (2.54-3.10)

Current use of			
Antiplatelet drugs	2451 (26.60)	7021 (15.88)	2.13 (2.02-2.26)
Oral anticoagulants	31 (0.34)	2242 (5.07)	0.06 (0.04-0.09)
Beta-Blockers	954 (10.35)	3837 (8.68)	1.23 (1.14-1.33)
Alfa-Blockers	233 (2.53)	960 (2.17)	1.20 (1.03-1.39)
ACEIs	1847 (20.05)	7571 (17.12)	1.31 (1.24-1.39)
ARBs	1553 (16.86)	6373 (14.41)	1.24 (1.16-1.32)
CCBs	1369 (14.86)	5215 (11.80)	1.37 (1.29-1.47)
Diuretics	1235 (13.40)	5596 (12.66)	1.12 (1.04-1.20)
Paracetamol	1462 (15.87)	6893 (15.59)	1.10 (1.02-1.18)
Metamizole	432 (4.69)	1590 (3.60)	1.41 (1.26-1.58)
NSAIDs	909 (9.87)	4197 (9.49)	1.11 (1.02-1.20)
Corticosteroids	188 (2.04)	715 (1.62)	1.28 (1.09-1.51)
Opioids	441 (4.79)	1677 (3.79)	1.30 (1.17-1.45)
CaS (with or without Vit D)	378 (4.10)	1788 (4.04)	1.00 (0.89-1.12)
Hormone replacement therapy [‡]	10 (0.11)	67 (0.15)	0.71 (0.36-1.39)
SERM	20 (0.22)	105 (0.24)	0.87 (0.54-1.41)
Strontium ranelate	18 (0.20)	57 (0.13)	1.47 (0.86-2.51)
Calcitonin	13 (0.14)	89 (0.20)	0.70 (0.39-1.25)
Denosumab	3 (0.03)	21 (0.05)	0.61 (0.18-2.04)
Teriparatide	6 (0.07)	24 (0.05)	1.12 (0.46-2.75)
PPIs	2795 (30.34)	11200 (25.33)	1.36 (1.28-1.44)
H_2 receptor blockers	226 (2.45)	736 (1.66)	1.50 (1.29-1.74)

Abbreviations: ACEIs: Angiotensin Converting Enzyme Inhibitors; ARBs: Angiotensin II-Receptor Blockers; BMI: Body Max Index; CaS: Calcium supplements; CCBs: Calcium-channel blockers; CI: Confidence Interval; COPD: chronic obstructive pulmonary disease; NSAIDs: Non-steroidal Anti-inflammatory Drugs; OR: Odds ratio; PPIs: Proton-pump inhibitors; SD: Standard Deviation; SERM: Selective estrogen receptor modulators.

[*] Adjusted only for matching factors (age, sex, and calendar year).
[†] Recorded as such, and/or when patients were using nitrates.
[§] Recorded as such, and/or when patients were using glucose-lowering drugs.
[⁋] Recorded as such, and/or when patients were using lipid-lowering drugs.
[‡] Including tibolone.

Supplementary Table 3. Risk of ischemic stroke associated with the ever use of oral bisphosphonates by duration, according to type of ischemic stroke (cardioembolic, non-cardioembolic).

**Overall**	**Cases (%) N=13781**	**Controls (%) N=65909**	**Non-adjusted OR* (95% CI)**	**Adjusted OR† (95% CI)**
Non-users	13127 (95.25)	63335 (96.09)	1 (Ref.)	1 (Ref.)
Ever users	654 (4.75)	2574 (3.91)	1.18 (1.08-1.29)	1.14 (1.02-1.27)
Duration <1 year	332 (2.41)	1394 (2.12)	1.12 (0.99-1.26)	1.06 (0.93-1.21)
Duration 1-3 years	165 (1.20)	668 (1.01)	1.14 (0.96-1.36)	1.13 (0.94-1.36)
Duration > 3 years	157 (1.14)	512 (0.78)	1.41 (1.17-1.69)	1.39 (1.14-1.69)
**BY PATHOPHYSIOLOGICAL SUBTYPE**				
**Cardioembolic stroke**	**Cases (%) N=4568**	**Controls (%) N=21697**	**Non-adjusted OR* (95% CI)**	**Adjusted OR† (95% CI)**
Non-users	4293 (93.98)	20744 (95.61)	1 (Ref.)	1 (Ref.)
Ever users	275 (6.02)	953 (4.39)	1.35 (1.17-1.55)	1.36 (1.14-1.63)
Duration <1 year	132 (2.89)	505 (2.33)	1.23 (1.01-1.50)	1.25 (1.00-1.57)
Duration 1-3 years	74 (1.62)	249 (1.15)	1.39 (1.06-1.81)	1.39 (1.03-1.88)
Duration > 3 years	69 (1.51)	199 (0.92)	1.59 (1.20-2.10)	1.65 (1.19-2.28)
**Non-cardioembolic stroke**	**Cases (%) N=9213**	**Controls (%) N=44212**	**Non-adjusted OR* (95% CI)**	**Adjusted OR† (95% CI)**
Non-users	8834 (95.89)	42591 (96.33)	1 (Ref.)	1 (Ref.)
Ever users	379 (4.11)	1621 (3.67)	1.08 (0.96-1.22)	1.03 (0.90-1.18)
Duration <1 year	200 (1.17)	889 (2.01)	1.05 (0.90-1.23)	0.99 (0.83-1.17)
Duration 1-3 years	91 (0.99)	419 (0.95)	1.00 (0.80-1.26)	0.99 (0.77-1.26)
Duration > 3 years	88 (0.96)	313 (0.71)	1.29 (1.02-1.65)	1.23 (0.95-1.60)

Abbreviations: CI: Confidence Interval; OR: Odds ratio.

*Adjusted only for matching factors (age, sex and calendar year).

†Adjusted for matching factors (age, sex and calendar year) plus number of primary care physician (PCP) visits in the last year, smoking, alcohol dependence (recorded as such by PCP), body mass index, transient ischemic attack, ischemic heart disease (including history of acute myocardial infarction or angina pectoris - recorded as such and/or use of nitrates), thromboembolism, heart failure, peripheral artery disease, hypertension, diabetes (recorded as such, and/or use of glucose-lowering drugs), dyslipidemia (registered as such, and/or use of lipid-lowering drugs), hyperuricemia (asymptomatic and gout), chronic obstructive pulmonary disease, rheumatoid arthritis, and chronic renal failure, and use within the last 30 days of antiplatelet agents, beta-blockers, alpha blockers, angiotensin-converting enzyme inhibitors, angiotensin II receptor antagonists, calcium antagonists, diuretics, paracetamol, metamizole, non-steroidal anti-inflammatory drugs, corticosteroids, opioids, calcium with/without vitamin D supplements, hormonal replacement therapy, estrogen receptor modulators, strontium ranelate, calcitonin, denosumab, teriparatide, proton pump inhibitors and H2-receptor antagonists.

Supplementary Table 4. Risk of ischemic stroke associated with the use of oral bisphosphonates by duration, according to type of ischemic stroke (cardioembolic, non-cardioembolic), including prevalent users.

**Overall**	**Cases (%) N=14322**	**Controls (%) N=71610**	**Non-adjusted OR* (95% CI)**	**Adjusted OR† (95% CI)**
Non-users	13127 (91.66)	65623 (91.64)	1 (Ref.)	1 (Ref.)
Current users	732 (5.11)	3735 (5.22)	0.98 (0.90-1.06)	0.97 (0.88-1.07)
Duration <1 year	253 (1.77)	1440 (2.01)	0.88 (0.77-1.01)	0.86 (0.74-1.00)
Duration ≥1 year	479 (3.34)	2295 (3.20)	1.04 (0.94-1.16)	1.04 (0.92-1.16)
1-3 years	249 (1.74)	1267 (1.77)	0.98 (0.85-1.13)	0.98 (0.84-1.14)
> 3 years	230 (1.61)	1028 (1.44)	1.12 (0.97-1.29)	1.11 (0.94-1.30)
Past users	463 (3.23)	2252 (3.14)	1.03 (0.93-1.14)	0.96 (0.86-1.08)
Time since discontinuation				
1-3 years	219 (1.53)	1273 (1.78)	0.86 (0.74-1.00)	0.83 (0.71-0.97)
> 3 years	244 (1.70)	979 (1.37)	1.25 (1.08-1.45)	1.14 (0.97-1.33)
**BY PATHOPHYSIOLOGICAL SUBTYPE**				
**Cardioembolic stroke**	**Cases (%) N=4780**	**Controls (%) N=23900**	**Non-adjusted OR* (95% CI)**	**Adjusted OR† (95% CI)**
Non-users	4293 (89.81)	21622 (90.47)	1 (Ref.)	1 (Ref.)
Current users	287 (6.00)	1392 (5.82)	1.04 (0.91-1.20)	1.06 (0.89-1.25)
Duration <1 year	94 (1.97)	510 (2.13)	0.93 (0.75-1.17)	0.90 (0.70-1.16)
Duration ≥1 year	193 (4.04)	882 (3.69)	1.11 (0.94-1.31)	1.15 (0.95-1.40)
1-3 years	106 (2.22)	477 (2.00)	1.13 (0.91-1.40)	1.14 (0.89-1.45)
> 3 years	87 (1.82)	405 (1.69)	1.09 (0.86-1.38)	1.16 (0.89-1.53)
Past users	200 (4.18)	886 (3.71)	1.15 (0.98-1.35)	1.15 (0.95-1.39)
Time since discontinuation				
1-3 years	86 (1.80)	488 (2.04)	0.89 (0.71-1.13)	0.93 (0.72-1.21)
> 3 years	114 (2.38)	398 (1.67)	1.46 (1.18-1.82)	1.43 (1.12-1.84)
**Non-cardioembolic stroke**	**Cases (%) N=9542**	**Controls (%) N=47710**	**Non-adjusted OR* (95% CI)**	**Adjusted OR† (95% CI)**
Non-users	8834 (92.58)	44001 (92.23)	1 (Ref.)	1 (Ref.)
Current users	445 (4.66)	2343 (4.91)	0.94 (0.85-1.05)	0.91 (0.81-1.04)
Duration <1 year	159 (1.67)	930 (1.95)	0.85 (0.72-1.01)	0.83 (0.69-1.00)
Duration ≥1 year	286 (3.00)	1413 (2.96)	1.00 (0.88-1.15)	0.97 (0.83-1.12)
1-3 years	143 (1.50)	790 (1.66)	0.90 (0.75-1.08)	0.88 (0.72-1.07)
> 3 years	143 (1.50)	623 (1.31)	1.14 (0.95-1.37)	1.07 (0.87-1.31)
Past users	263 (2.76)	1366 (2.86)	0.95 (0.83-1.09)	0.89 (0.77-1.04)
Time since discontinuation				
1-3 year	133 (1.39)	785 (1.65)	0.84 (0.70-1.01)	0.82 (0.67-1.00)
> 3 years	130 (1.36)	581 (1.22)	1.11 (0.92-1.35)	0.99 (0.80-1.22)

Abbreviations: CI: Confidence Interval; OR: Odds ratio.
*Adjusted only for matching factors (age, sex and calendar year).
†Adjusted for matching factors (age, sex and calendar year) plus number of primary care physician (PCP) visits in the last year, smoking, alcohol dependence (recorded as such by PCP), body mass index, transient ischemic attack, ischemic heart disease (including history of acute myocardial infarction or angina pectoris - recorded as such and/or use of nitrates), thromboembolism, heart failure, peripheral artery disease, hypertension, diabetes (recorded as such, and/or use of glucose-lowering drugs), dyslipidemia (registered as such, and/or use of lipid-lowering drugs), hyperuricemia (asymptomatic and gout), chronic obstructive pulmonary disease, rheumatoid arthritis, and chronic renal failure, and use within the last 30 days of antiplatelet agents, beta-blockers, alpha blockers, angiotensin-converting enzyme inhibitors, angiotensin II receptor antagonists, calcium antagonists, diuretics, paracetamol, metamizole, non-steroidal anti-inflammatory drugs, corticosteroids, opioids, calcium with/without vitamin D supplements, hormonal replacement therapy, estrogen receptor modulators, strontium ranelate, calcitonin, denosumab, teriparatide, proton pump inhibitors and H2-receptor antagonists.

Supplementary Table 5. Estimated population impact of the risk of cardioembolic ischemic stroke associated with long-term use of oral bisphosphonates (5 years).

**Standardized Annual incidence of IS (per 100,000 persons)** (GBD 2019 Stroke Collaborators 2021)	**Estimated annual incidence of cardioembolic IS (33% of all cases) (per 100,000 persons)**	**5-year cumulative incidence of cardioembolic IS (per 100,000 persons)**	**AOR associated with > 3 years use of bisphosphonates**	**5-year cumulative incidence of cardioembolic IS among exposed to bisphosphonates (per 100,000 persons)**	**AR among the exposed (per 100,000 persons)**	**NNH**
94	31	155	1.81	281	126	796
94	31	155	1.25 (LLCI)	194	39	2581
94	31	155	2.62 (ULCI)	406	251	398

Abbreviations: AR: Attributable Risk; AOR: Adjusted odds ratio; CI: Confidence Interval; IS: Ischemic Stroke; LLCI: Lower level CI; NNH: Number Needed to Harm; ULCI: Upper level CI.

REFERENCES

GBD 2019 Stroke Collaborators (2021). Global, regional, and national burden of stroke and its risk factors, 1990-2019: a systematic analysis for the Global Burden of Disease Study 2019. Lancet Neurol. 20, 795-820. doi:10.1016/S1474-4422(21)00252-0.

Maciá-Martínez, M. A., Gil, M., Huerta, C., Martín-Merino, E., Álvarez, A., Bryant, V., et al. (2020). Base de Datos para la Investigación Farmacoepidemiológica en Atención Primaria (BIFAP): A data resource for pharmacoepidemiology in Spain. Pharmacoepidemiol Drug Saf. 29, 1236-1245. doi:10.1002/pds.5006.

Rodríguez-Martín, S., González-Bermejo, D., Rodríguez-Miguel, A., Barreira, D., García-Lledó, A., Gil, M., et al. (2020). Risk of Myocardial Infarction Among New Users of Calcium Supplements Alone or Combined With Vitamin D: A Population-Based Case-Control Study. *Clin Pharmacol Ther*. 107, 359-368. doi: 10.1002/cpt.1636.
